# Dynamic frailty and depressive symptoms in relation to incident stroke: findings from five harmonized longitudinal cohorts

**DOI:** 10.3389/fneur.2026.1880619

**Published:** 2026-07-14

**Authors:** Yicheng Jiang, Qi Wang, Jinsen Zhang, Ming Shan, Qinglong Guo

**Affiliations:** 1 Department of Neurosurgery, The First Affiliated Hospital of Anhui Medical University, Hefei, China; 2 Epilepsy and Functional Neurological Disorders Brain–Computer Interface Specialized Diagnosis and Treatment Center, The First Affiliated Hospital of Anhui Medical University, Hefei, China; 3 Department of Biomedical Engineering, College of Engineering, Boston University, Boston, MA, United States; 4 Department of Neurosurgery, Huashan Hospital, Shanghai Medical College, Fudan University, Shanghai, China

**Keywords:** aging cohorts, competing risk, depressive symptoms, frailty index, incident stroke, mediation analysis, restricted cubic spline, two-wave cross-lagged panel model

## Abstract

**Background:**

Frailty and depressive symptoms are common in later life and may be related to cerebrovascular risk. Evidence remains limited on whether frailty burden and frailty change are associated with incident stroke across diverse aging cohorts.

**Methods:**

We analyzed harmonized longitudinal data from five population-based aging cohorts: the Health and Retirement Study (HRS), China Health and Retirement Longitudinal Study (CHARLS), Survey of Health, Ageing and Retirement in Europe (SHARE), English Longitudinal Study of Ageing (ELSA), and Mexican Health and Aging Study (MHAS). Frailty was measured using a harmonized 24-item deficit-accumulation frailty index (FI). The primary analysis used cohort-specific Cox proportional hazards models to estimate associations between baseline FI and first observed incident stroke during follow-up. Secondary exploratory analyses evaluated nonlinearity, FI change, competing mortality, depressive symptoms as a pathway marker, and two-wave cross-lagged associations.

**Results:**

The analytic sample included 81482 participants and 5,089 incident stroke events. In fully adjusted cohort-specific Cox models, each 0.1-unit increase in FI was associated with higher stroke risk in HRS, CHARLS, SHARE, and MHAS, but not in ELSA. Substantial between-cohort heterogeneity was observed; therefore, cohort-specific estimates were interpreted as the primary results and the random-effects pooled estimate was treated as descriptive. Fine-Gray sensitivity analyses treating death as a competing event supported positive frailty-stroke associations across all five cohorts. Restricted cubic spline (RCS) analyses suggested nonlinear associations for baseline FI and FI change. Exploratory pathway analyses indicated that depressive symptoms statistically accounted for part of selected frailty-stroke associations, although patterns varied by cohort and exposure definition. Two-wave cross-lagged panel models (CLPMs) suggested small, cohort-specific prospective associations between elevated frailty vulnerability and later depressive symptoms or stroke; these findings were interpreted as exploratory temporal associations rather than causal within-person effects.

**Conclusion:**

Higher frailty burden was associated with incident stroke in most, but not all, harmonized aging cohorts, with substantial heterogeneity across populations. The findings support repeated frailty assessment and integrated mood evaluation in older adults while emphasizing the need for cohort-specific interpretation and confirmatory studies with adjudicated stroke outcomes.

## 1 Introduction

Stroke remains a leading cause of death and long-term disability worldwide, particularly among middle-aged and older adults (1, 2). In addition to established vascular risk factors, geriatric syndromes such as frailty may capture biological vulnerability that is not fully represented by chronological age or single comorbidities (3, 4).

Frailty is commonly conceptualized using both the physical phenotype and the deficit-accumulation frailty index (FI) (5–7). The FI is particularly suitable for large population-based cohorts because it summarizes multiple health deficits across chronic disease, symptoms, sensory problems, and functional limitations. Prior studies indicate that frailty is associated with adverse cardiovascular and cerebrovascular outcomes, including stroke incidence and post-stroke prognosis (8–10).

Frailty is not a fixed state. Older adults may remain robust, deteriorate from robustness to frailty, remain persistently frail, or recover from frailty to a less vulnerable state (11–13). Such transitions may have different prognostic meanings from a single baseline FI value. In parallel, depressive symptoms are associated with stroke risk and often co-occur with frailty (14, 15). Physical deterioration may worsen mood through loss of independence, pain, social isolation, and functional limitation, while depression may aggravate vascular risk through inflammation, autonomic dysfunction, inactivity, poor diet, and medication non-adherence (16–20).

We used harmonized data from the Health and Retirement Study (HRS), China Health and Retirement Longitudinal Study (CHARLS), Survey of Health, Ageing and Retirement in Europe (SHARE), English Longitudinal Study of Ageing (ELSA), and Mexican Health and Aging Study (MHAS) to address related but distinct questions. Cox models evaluated the primary association between baseline frailty burden and incident stroke. Frailty-change analyses examined whether worsening or improvement in frailty vulnerability was associated with subsequent stroke risk. Exploratory pathway analyses assessed whether depressive symptoms statistically accounted for part of the association between frailty-related exposures and stroke. Finally, exploratory two-wave CLPMs evaluated temporal ordering and reciprocal longitudinal associations among elevated frailty vulnerability, depressive symptoms, and stroke status. Because the study was observational and not preregistered, secondary and pathway analyses were interpreted as exploratory and hypothesis-generating rather than causal.

## 2 Methods

### 2.1 Study design and cohorts

This longitudinal multi-cohort study used harmonized data from five population-based aging studies: HRS in the United States, CHARLS in China, SHARE in Europe and Israel, ELSA in England, and MHAS in Mexico. These studies participate in the Gateway to Global Aging Data initiative, which harmonizes variables to facilitate cross-national comparisons of aging and health (21–26).

**Figure 1:**
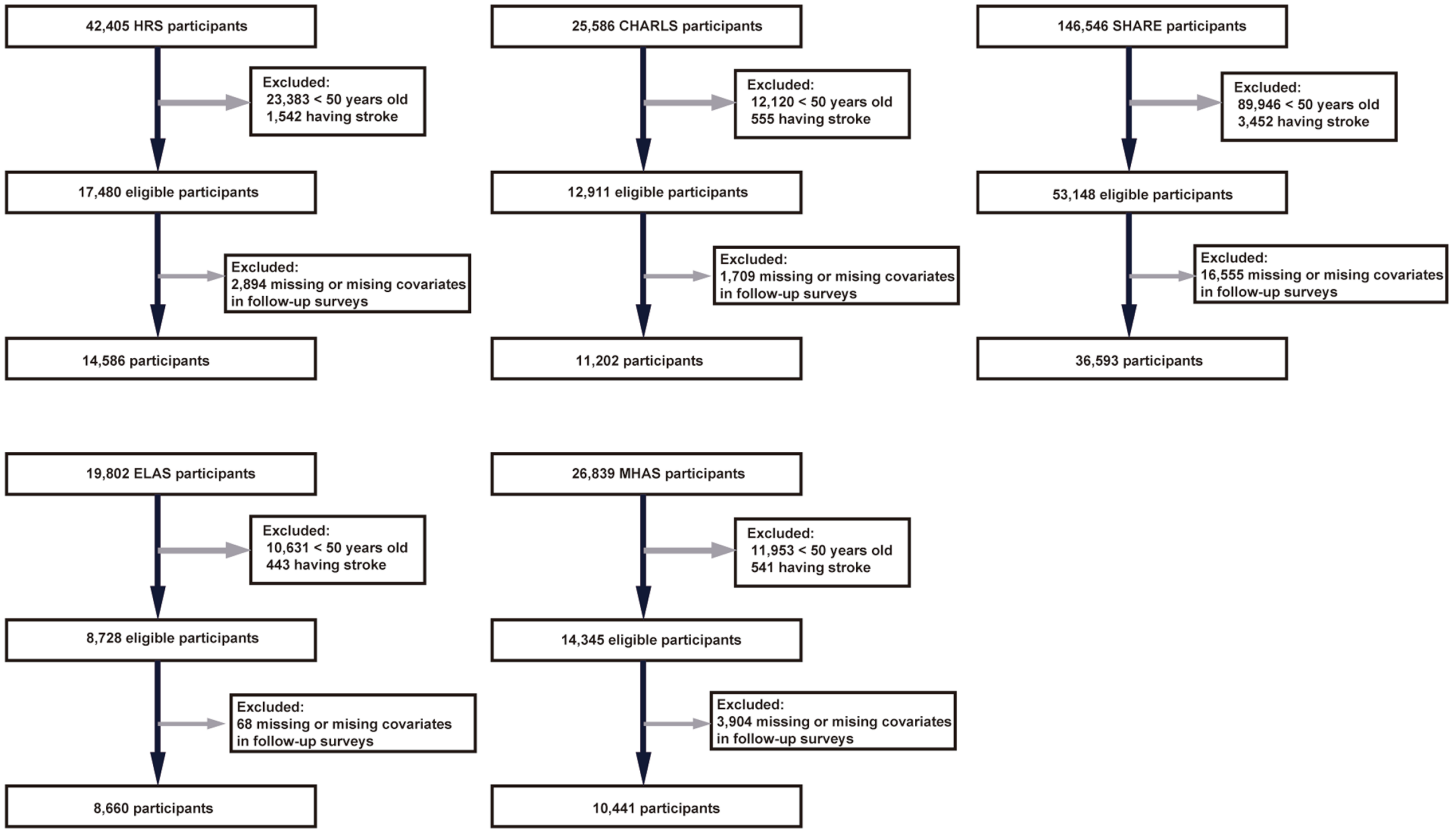
Study flowchart. Participant selection and analytic sample construction across HRS, CHARLS, SHARE, ELSA, and MHAS.

The analytic sample was constructed separately within each cohort before harmonized analyses. Baseline was defined as the first survey wave with harmonized information on frailty, depressive symptoms, covariates, and subsequent stroke follow-up. The primary baseline analyses used HRS wave 5, CHARLS wave 1, SHARE wave 4, ELSA wave 2, and MHAS wave 1. Participants were eligible if they participated in the cohort-specific baseline wave, had no self-reported history of stroke at baseline, had a calculable baseline FI, and had available follow-up information for incident stroke. Participants were excluded if they had prevalent stroke at baseline, missing or invalid baseline FI, missing incident-stroke status or follow-up time, or missing key covariates required for a given adjusted model. Cohort-specific survey schedules and analytic sample construction are summarized in Figure 1 and Supplementary Table S1.

The primary repeated-measure interval used for frailty-change analyses was based on adjacent available survey waves. HRS, CHARLS, SHARE, and ELSA were conducted at approximately biennial intervals, whereas MHAS had a variable multi-year survey structure. Therefore, the T1-to-T2 frailty-change interval was defined within each cohort according to its survey schedule rather than as a uniform calendar interval across all cohorts.

### 2.2 Analytic framework

The analyses were organized to move from descriptive validation of the harmonized FI to estimation of the primary frailty-stroke association, followed by exploration of heterogeneity, nonlinearity, frailty change, competing mortality, and potential psychological pathways. First, we evaluated within-cohort FI distributions and age gradients. Second, cohort-specific Cox proportional hazards models estimated the association between baseline FI and incident stroke. Third, sequential adjustment and subgroup analyses evaluated robustness across demographic, socioeconomic, social, behavioral, and clinical covariates. Fourth, RCS models assessed nonlinear associations for baseline FI and FI change. Fifth, exploratory pathway analyses assessed whether depressive symptoms statistically accounted for part of selected frailty-stroke associations. Sixth, competing-risk and proportional-hazards diagnostics evaluated sensitivity to death before stroke and model assumptions. Finally, a two-wave CLPM explored reciprocal temporal associations among elevated frailty vulnerability, depressive symptoms, and stroke status.

### 2.3 Frailty index

Frailty was measured using a 24-item FI based on the deficit-accumulation approach (6, 7). Deficits covered chronic diseases, sensory and physical symptoms, mobility limitations, activities of daily living, instrumental activities of daily living, self-rated health, and body mass index deficits. The complete list of items and cross-cohort harmonization rules is provided in Supplementary Table S1. Cohort-specific source variables were mapped to the same 24 conceptual deficit domains using predefined harmonization rules. When a source variable could not be validly constructed for a participant or wave, that item was treated as missing and incorporated into the FI calculation only through the prorating denominator.

Within each cohort and wave, the FI was calculated using a prorating approach. Specifically, the numerator was the number of observed deficits and the denominator was the number of non-missing FI items. The FI was calculated only when at least 80% of the 24 items were available, corresponding to at least 19 observed items. Participants with fewer than 19 observed items at a given wave were assigned missing FI for that wave. This prorating rule was applied consistently across HRS, CHARLS, SHARE, ELSA, and MHAS. Missing items were not coded as zero, were not treated as absent deficits, and were not imputed. For categorical analyses of established frailty, FI > 0.25 was used to define frail status, and FI ≤ 0.25 was used to define non-frail status. For exploratory mediation-related and two-wave cross-lagged analyses, FI ≥ 0.10 was used to indicate elevated frailty vulnerability or early deficit accumulation rather than established frailty.

### 2.4 Incident stroke

The primary outcome was first observed incident stroke during follow-up. Incident stroke was defined as the first post-baseline report of physician-diagnosed stroke among participants who were free of self-reported stroke at baseline. Harmonized self-reported physician diagnosis was supplemented, where available, by proxy interviews, exit interviews, linked records, or cohort-specific follow-up sources. Participants without stroke were censored at death, loss to follow-up, or the final available survey wave, whichever occurred first. Recurrent stroke events and stroke subtypes were not modeled because harmonized recurrent-event and subtype information was not consistently available across cohorts.

### 2.5 Depressive symptoms and covariates

Depressive symptoms were assessed using cohort-specific validated instruments: the 8-item Center for Epidemiologic Studies Depression Scale in HRS and ELSA, the 10-item CES-D in CHARLS and MHAS, and the EURO-D scale in SHARE (27). Scores were standardized within cohort to support cross-cohort comparability. Binary depressive-symptom status was defined using established cohort-specific cut-points in exploratory mediation-related and cross-lagged analyses.

Covariates were selected *a priori* based on known or plausible associations with frailty and incident stroke. They included age, sex, education, employment status, marital status, children, smoking, drinking, social activity, physical activity, hypertension, diabetes, heart disease, and depressive symptoms when depressive symptoms were not modeled as a pathway marker. Social activity was harmonized as any participation versus no participation in comparable social, community, volunteer, club, or group activities when available in the harmonized cohort data. Physical activity was harmonized as active versus inactive using cohort-specific items that captured regular moderate or vigorous physical activity or comparable activity-frequency indicators. Because source questions differed across cohorts, these variables were treated as broad harmonized indicators rather than identical measures. Covariates were measured at the cohort-specific baseline wave unless otherwise stated. Operational definitions, timing, and rationale are provided in Supplementary Table S2. Model-specific complete-case analysis was used for non-FI covariates.

### 2.6 Statistical analysis

Baseline characteristics were summarized by cohort as means with standard deviations for continuous variables and counts with percentages for categorical variables. Cox proportional hazards models estimated hazard ratios (HRs) for first observed incident stroke per 0.1-unit increase in baseline FI. Sequential models were used to evaluate robustness to progressive adjustment. Model 1 adjusted for age and sex. Model 2 additionally adjusted for socioeconomic factors. Model 3 additionally adjusted for family and social factors. Model 4 further adjusted for behavioral and clinical factors, according to harmonized variable availability. Cohort-specific estimates were interpreted as the primary results. Random-effects pooled estimates were treated as descriptive because of substantial between-cohort heterogeneity.

The proportional-hazards assumption was assessed using scaled Schoenfeld residual tests for the frailty term and global model (28, 29). For each cohort-specific Cox model, test statistics, degrees of freedom, nominal *p* values, and false-discovery-rate (FDR)-adjusted Q values were reported. Smoothed scaled Schoenfeld residual plots were also inspected to evaluate the direction and timing of departures from proportionality. Time-varying coefficient models and stratified Cox approaches were considered as alternative strategies. These models were not used as the primary approach because the study aimed to maintain the same harmonized modeling structure across cohorts and to facilitate comparison of cohort-specific estimates. Instead, the prespecified Cox models were retained and supplemented with proportional-hazards diagnostics, RCS analyses, and Fine-Gray competing-risk sensitivity analyses. RCS models evaluated potential nonlinear associations for baseline FI and FI change (28, 29). Subgroup analyses evaluated consistency across sex, age, education, employment, marital status, smoking, drinking, and physical activity strata.

Fine-Gray competing-risk regression was used as a sensitivity analysis with death before incident stroke treated as the competing event. The primary competing-risk sensitivity model adjusted for age and sex; more extensively adjusted models were reported as exploratory because available covariates and effective sample sizes differed across cohorts.

Exploratory mediation-related analyses evaluated depressive symptoms as a pathway marker rather than a definitive causal mediator (30–32). The baseline frailty analysis was treated as an exploratory cross-sectional statistical decomposition because baseline FI and depressive symptoms were measured at the same survey wave; therefore, temporal ordering between frailty and depressive symptoms was not established and causal mediation was not inferred. Dynamic mediation-related analyses defined frailty increase as transition from FI < 0.10 at T1 to FI ≥ 0.10 at T2 and frailty improvement as transition from FI ≥ 0.10 at T1 to FI < 0.10 at T2, where T1 and T2 represented adjacent survey waves within each cohort. Depressive symptoms were assessed after the frailty-change interval, and incident stroke was followed subsequently. The frailty-change models were not additionally adjusted for the exact T1-to-T2 interval duration because this interval was determined by the cohort-specific survey design and was largely fixed within each cohort. Follow-up time for incident stroke after the frailty-change interval was accounted for in the time-to-event framework. Frailty change was modeled as a time-fixed derived exposure rather than as a repeatedly updated time-varying covariate. The exposure was calculated over a prespecified adjacent-wave interval within each cohort, from T1 to T2, and was then used to predict subsequent incident stroke. This approach was selected to preserve temporal ordering between the frailty-change exposure, subsequent depressive symptoms, and later incident stroke, and to improve comparability across cohorts with different repeated-measure structures. No extended Cox model with time-updated frailty values was fitted. The FI ≥ 0.10 threshold was used only for exploratory pathway and two-wave CLPM analyses and does not define established frailty; therefore, elevated frailty vulnerability was used consistently rather than established frailty terminology.

The two-wave CLPM was employed because the harmonized three-variable analysis used two adjacent waves for each cohort (33, 34). The model estimated autoregressive paths, cross-lagged paths, baseline correlations, and residual correlations at follow-up. It was interpreted as an exploratory temporal association analysis and not as a within-person/between-person decomposition. Multiple-testing correction was performed using the Benjamini-Hochberg FDR procedure with statistical significance defined as *q* < 0.05. FDR correction was applied separately within prespecified families of tests, including cohort-specific Cox model tests, subgroup Cox analyses, RCS overall and nonlinear tests, mediation-related indirect-effect tests, two-wave CLPM paths, proportional-hazards diagnostics, and competing-risk sensitivity analyses.

## 3 Results

### 3.1 Study population and baseline characteristics

After exclusions shown in Figure 1, the final main Cox analytic sample included 81482 participants across five cohorts. SHARE contributed the largest sample (*n* = 36,593), followed by HRS (*n* = 14,586), CHARLS (*n* = 11,202), MHAS (*n* = 10,441), and ELSA (*n* = 8,660). Incident stroke events were observed in 1,841 HRS participants, 828 CHARLS participants, 1,867 SHARE participants, 378 ELSA participants, and 175 MHAS participants. Baseline characteristics, follow-up duration, and incident stroke events are shown by cohort in Table 1.

**Table 1:** Baseline characteristics, follow-up duration, and incident stroke events by cohort.

Characteristic	HRS	CHARLS	SHARE	ELSA	MHAS
Demographic characteristics					
Age, years	65.91 (9.25)	61.39 (7.92)	64.83 (9.21)	65.85 (9.82)	64.07 (8.84)
Sex					
Female	8,615 (59.06%)	5,715 (51.02%)	21,103 (57.67%)	4,823 (55.69%)	6,058 (58.02%)
Male	5,971 (40.94%)	5,487 (48.98%)	15,490 (42.33%)	3,837 (44.31%)	4,383 (41.98%)
Socioeconomic characteristics					
Education					
Below secondary education	3,605 (24.72%)	1,148 (10.25%)	9,915 (27.10%)	2,987 (34.49%)	3,212 (30.76%)
Secondary education or above	10,981 (75.28%)	10,054 (89.75%)	26,678 (72.90%)	5,673 (65.51%)	7,229 (69.24%)
Employment status					
Not currently employed	9,754 (66.87%)	1823 (16.27%)	24,868 (67.96%)	7,738 (89.35%)	1,448 (13.87%)
Currently employed	4,832 (33.13%)	9,379 (83.73%)	11,725 (32.04%)	922 (10.65%)	8,993 (86.13%)
Family and social characteristics					
Marital status					
Not partnered	4,400 (30.17%)	2,539 (22.67%)	13,894 (37.97%)	3,857 (44.54%)	4,198 (40.21%)
Married or cohabiting	10,186 (69.83%)	8,663 (77.33%)	22,699 (62.03%)	4,803 (55.46%)	6,243 (59.79%)
Children					
No children	1,024 (7.02%)	103 (0.92%)	2,607 (7.12%)	906 (10.46%)	318 (3.05%)
One or more children	13,562 (92.98%)	11,099 (99.08%)	33,986 (92.88%)	7,754 (89.54%)	10,123 (96.95%)
Social activity					
No social activity	Not available	6,248 (55.78%)	18,851 (51.52%)	5,960 (68.82%)	2043 (19.57%)
Any social activity	Not available	4,954 (44.22%)	17,742 (48.48%)	2,700 (31.18%)	8,398 (80.43%)
Behavioral characteristics					
Smoking status					
Never or former smoker	12,434 (85.25%)	6,619 (59.09%)	20,059 (54.82%)	3,206 (37.02%)	6,688 (64.06%)
Current smoker	2,152 (14.75%)	4,583 (40.91%)	16,534 (45.18%)	5,454 (62.98%)	3,753 (35.94%)
Drinking status					
Non-drinker	7,553 (51.78%)	6,514 (58.15%)	7,463 (20.39%)	1893 (21.86%)	7,666 (73.42%)
Current drinker	7,033 (48.22%)	4,688 (41.85%)	29,130 (79.61%)	6,767 (78.14%)	2,775 (26.58%)
Physical activity					
Inactive	5,158 (35.36%)	3,661 (32.68%)	29,857 (81.59%)	2,254 (26.03%)	3,985 (38.17%)
Active	9,428 (64.64%)	7,541 (67.32%)	6,736 (18.41%)	6,406 (73.97%)	6,456 (61.83%)
Clinical and frailty characteristics					
Frailty status					
Non-frail (FI ≤ 0.25)	10,644 (72.97%)	9,889 (88.28%)	32,215 (88.04%)	7,258 (83.81%)	8,081 (77.40%)
Frail (FI > 0.25)	3,942 (27.03%)	1,313 (11.72%)	4,378 (11.96%)	1,402 (16.19%)	2,360 (22.60%)
Follow-up information and incident stroke events					
Follow-up duration, years	13.00 (5.02)	6.59 (1.02)	6.92 (1.74)	13.75 (1.35)	6.02 (1.5)
Incident stroke during follow-up					
No incident stroke	12,745 (87.38%)	10,374 (92.61%)	34,726 (94.90%)	8,282 (95.64%)	10,266 (98.32%)
Incident stroke	1841 (12.62%)	828 (7.39%)	1867 (5.10%)	378 (4.36%)	175 (1.68%)

Continuous variables are presented as mean (standard deviation), and categorical variables are presented as frequency (percentage). Baseline variables were measured at the cohort-specific baseline wave unless otherwise stated. Follow-up duration refers to time from baseline to first observed incident stroke, death, loss to follow-up, or the final available survey wave. Physical activity and social activity were harmonized across cohorts where comparable indicators were available. Social activity was categorized as any social/community participation versus no social/community participation where comparable harmonized indicators were available. ‘Not available’ indicates that a comparable harmonized social-activity variable was not available for that cohort and should not be interpreted as no activity or as individual-level missingness. Physical activity was categorized as active versus inactive using cohort-specific harmonized indicators of regular physical activity. Frailty status was defined using FI >0.25 for established frailty. Incident stroke refers to the first observed post-baseline stroke report during follow-up among participants free of stroke at baseline.

**Figure 2:**
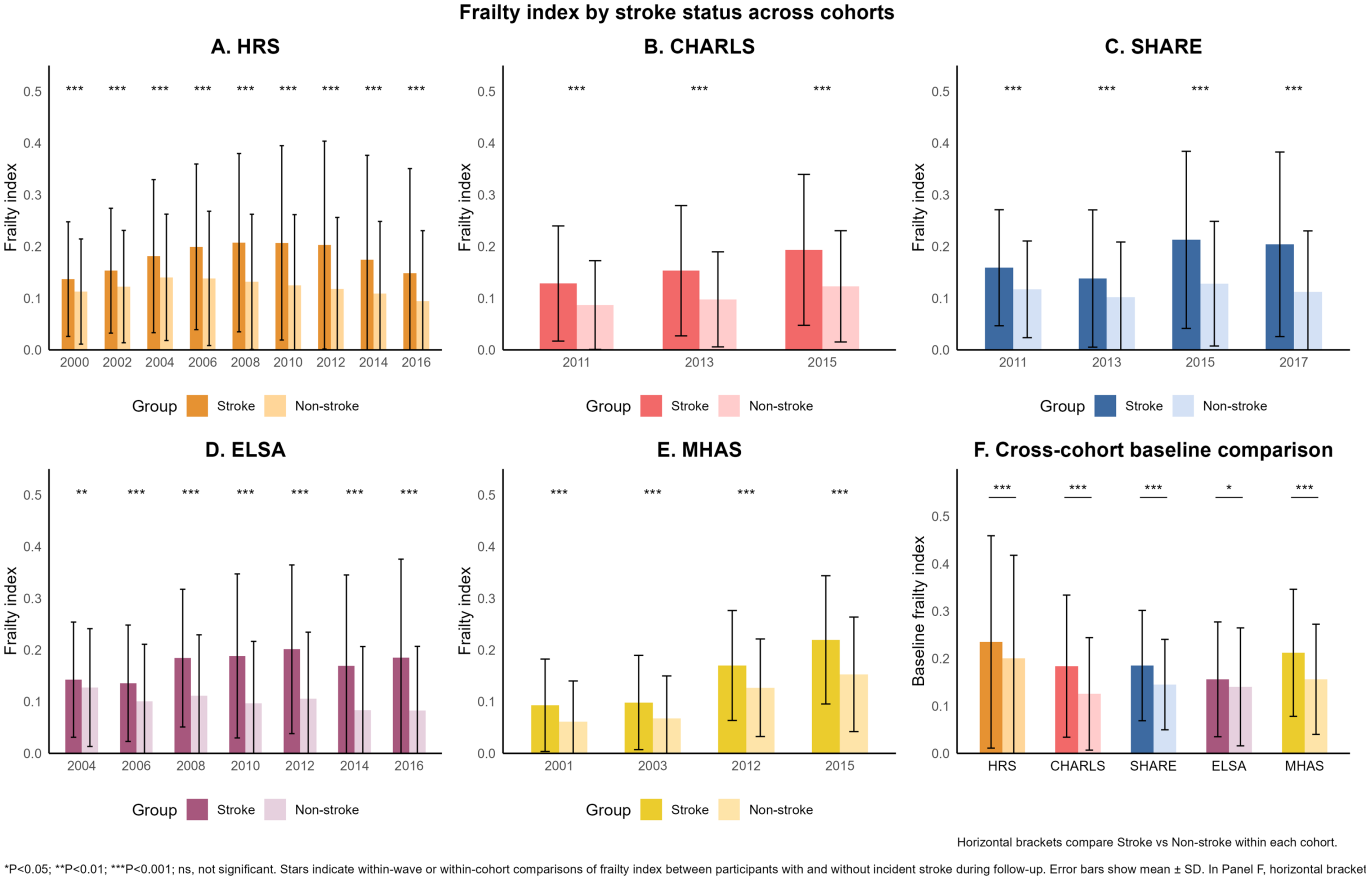
Frailty index by stroke status across survey years and cohorts, including cross-cohort baseline comparison. Error bars represent standard deviations; significance stars denote between-group comparisons. **(A)** HRS; **(B)** CHARLS; **(C)** SHARE; **(D)** ELSA; **(E)** MHAS; and **(F)** cross-cohort comparison of baseline frailty index by stroke status.

Participants who developed stroke generally had higher FI values than those who remained stroke-free across survey waves (Figure 2). Descriptive supplementary analyses showed right-skewed FI distributions and positive age gradients within each cohort, supporting the expected distributional properties of the harmonized FI (Supplementary Figures S1–S3; Supplementary Tables S2–S5).

### 3.2 Baseline frailty index and incident stroke in Cox and competing-risk models

Table 2 presents cohort-specific Cox proportional hazards models for the association between baseline FI and first observed incident stroke. In the fully adjusted model, each 0.1-unit increase in FI was associated with higher incident stroke risk in HRS (HR 1.07, 95% CI 1.05–1.09), CHARLS (HR 1.35, 95% CI 1.29–1.41), SHARE (HR 1.30, 95% CI 1.25–1.35), and MHAS (HR 1.31, 95% CI 1.17–1.46). The scaled Schoenfeld residual tests for the frailty index term showed no evidence of non-proportionality in HRS (*χ*^2^ = 0.22, *p* = 0.637), SHARE (*χ^2^* = 0.01, *p* = 0.939), or MHAS (*χ*^2^ = 0.65, *p* = 0.422). Nominal evidence of non-proportionality for the frailty term was observed in CHARLS (*χ*^2^ = 4.55, *p* = 0.033) and ELSA (*χ*^2^ = 4.89, *p* = 0.027), although these frailty-term tests did not remain significant after FDR correction (Supplementary Table S12). Global tests were significant for CHARLS and ELSA, indicating broader model-level departures from proportionality. Visual inspection of the smoothed Schoenfeld residual curves suggested that the frailty association in these cohorts varied over follow-up and tended to attenuate over time. The corresponding association in ELSA was not statistically significant (HR 1.01, 95% CI 0.93–1.10). Because between-cohort heterogeneity was substantial, cohort-specific estimates were interpreted as the primary results; the fully adjusted forest plot is provided as Supplementary Figure S4.

**Table 2:** Cohort-specific Cox proportional hazards models for the association between baseline frailty index and first observed incident stroke during follow-up.

Cohort	*N*	Events	Model 1 HR (95% CI)	*p*	Model 2 HR (95% CI)	*p*	Model 3 HR (95% CI)	*p*	Model 4 HR (95% CI)	*p*
HRS	14,586	1841	1.08 (1.06–1.10)	<0.001	1.08 (1.06–1.10)	<0.001	1.08 (1.06–1.10)	<0.001	1.07 (1.05–1.09)	<0.001
CHARLS	11,202	828	1.35 (1.30–1.41)	<0.001	1.35 (1.29–1.41)	<0.001	1.35 (1.29–1.41)	<0.001	1.35 (1.29–1.41)	<0.001
SHARE	36,593	1867	1.33 (1.28–1.38)	<0.001	1.31 (1.26–1.36)	<0.001	1.31 (1.26–1.36)	<0.001	1.30 (1.25–1.35)	<0.001
ELSA	8,660	378	1.02 (0.95–1.11)	0.550	1.02 (0.94–1.11)	0.627	1.02 (0.94–1.10)	0.651	1.01 (0.93–1.10)	0.827
MHAS	10,441	175	1.32 (1.19–1.47)	<0.001	1.32 (1.19–1.47)	<0.001	1.33 (1.19–1.48)	<0.001	1.31 (1.17–1.46)	<0.001

HR, hazard ratio; CI, confidence interval; FI, frailty index. HRs are reported per 0.1-unit increase in baseline FI. The outcome was first observed incident stroke during follow-up among participants free of stroke at baseline. Model 1 adjusted for age and sex. Model 2 additionally adjusted for socioeconomic factors. Model 3 additionally adjusted for family and social factors. Model 4 additionally adjusted for behavioral and clinical factors according to harmonized variable availability. *p* values are two-sided.

**Figure 3:**
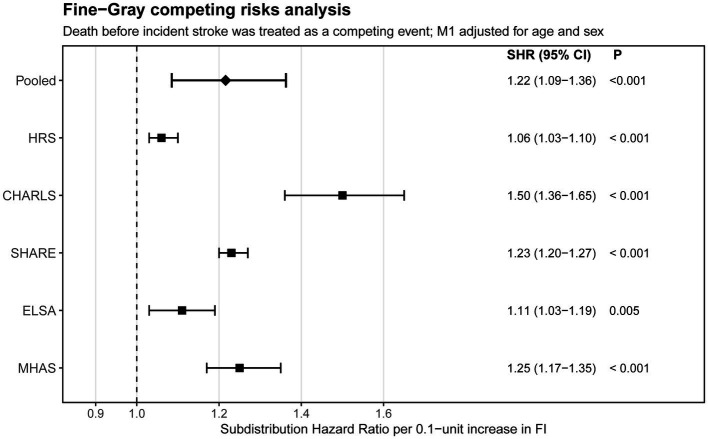
Fine-Gray competing-risk sensitivity analysis of baseline frailty index and incident stroke. Death before incident stroke was treated as a competing event.

Fine-Gray analyses treating death as a competing event supported the robustness of the frailty-stroke association (Figure 3; Supplementary Figure S5; Supplementary Tables S6–S11). In the age- and sex-adjusted competing-risk model, the random-effects pooled subdistribution hazard ratio (sHR) per 0.1-unit FI increase was 1.22 (95% CI, 1.09–1.36), and the ELSA-specific sHR was 1.11 (95% CI, 1.03–1.19; *p* = 0.005). Exploratory M2-M4 Fine-Gray models were broadly consistent but were interpreted cautiously because effective sample sizes and covariate availability varied across cohorts. Cohort-specific sHRs were positive across all five cohorts.

The difference between the null Cox estimate and the positive Fine-Gray estimate in ELSA may reflect the different estimands of the two models. Cox models estimate cause-specific hazards among participants who remain event-free and censor deaths before stroke, whereas Fine-Gray models estimate the subdistribution hazard for the cumulative incidence of stroke while retaining deaths as competing events.

Because proportional-hazards departures were observed in CHARLS and ELSA, their Cox hazard ratios should be interpreted as average associations over follow-up rather than strictly time-constant hazard ratios.

### 3.3 Subgroup and stroke-free survival analyses

**Figure 4:**
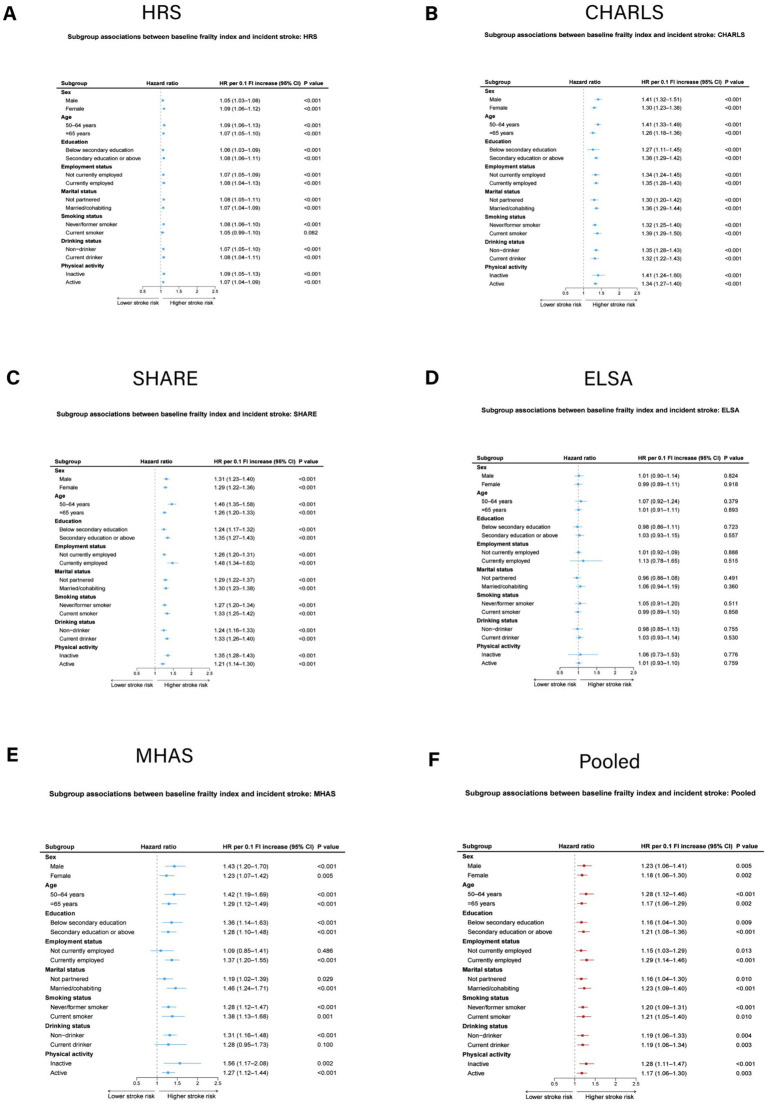
Subgroup associations between baseline frailty index and first observed incident stroke. **(A)** HRS; **(B)** CHARLS; **(C)** SHARE; **(D)** ELSA; **(E)** MHAS; and **(F)** pooled analysis.

Subgroup analyses (Figure 4) showed broadly positive associations between FI and incident stroke across sex, age, education, employment, marital status, smoking, drinking, and physical activity strata. Precision varied across cohorts and subgroups, but the overall pattern did not suggest that the association was limited to a single demographic or behavioral subgroup.

**Figure 5:**
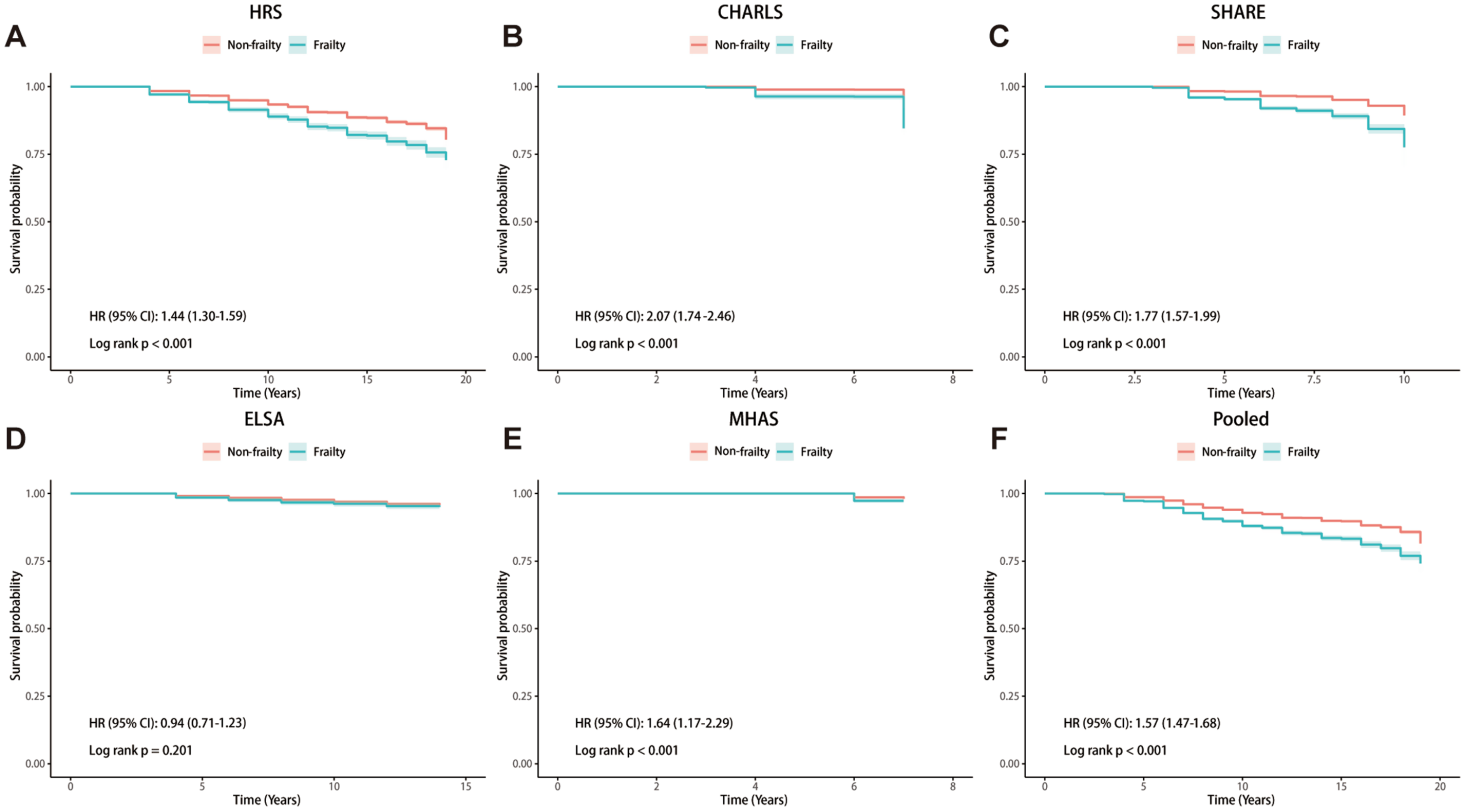
Kaplan–Meier curves for stroke-free survival by frailty status across cohorts and the pooled sample. **(A)** HRS; **(B)** CHARLS; **(C)** SHARE; **(D)** ELSA; **(E)** MHAS; and **(F)** pooled analysis.

Kaplan–Meier curves (Figure 5) indicated lower stroke-free survival among frail participants in HRS, CHARLS, SHARE, MHAS, and the pooled sample. Separation by frailty status was less evident in ELSA, consistent with the attenuated Cox estimate in that cohort.

### 3.4 Nonlinear associations of baseline FI and FI change with incident stroke

**Figure 6:**
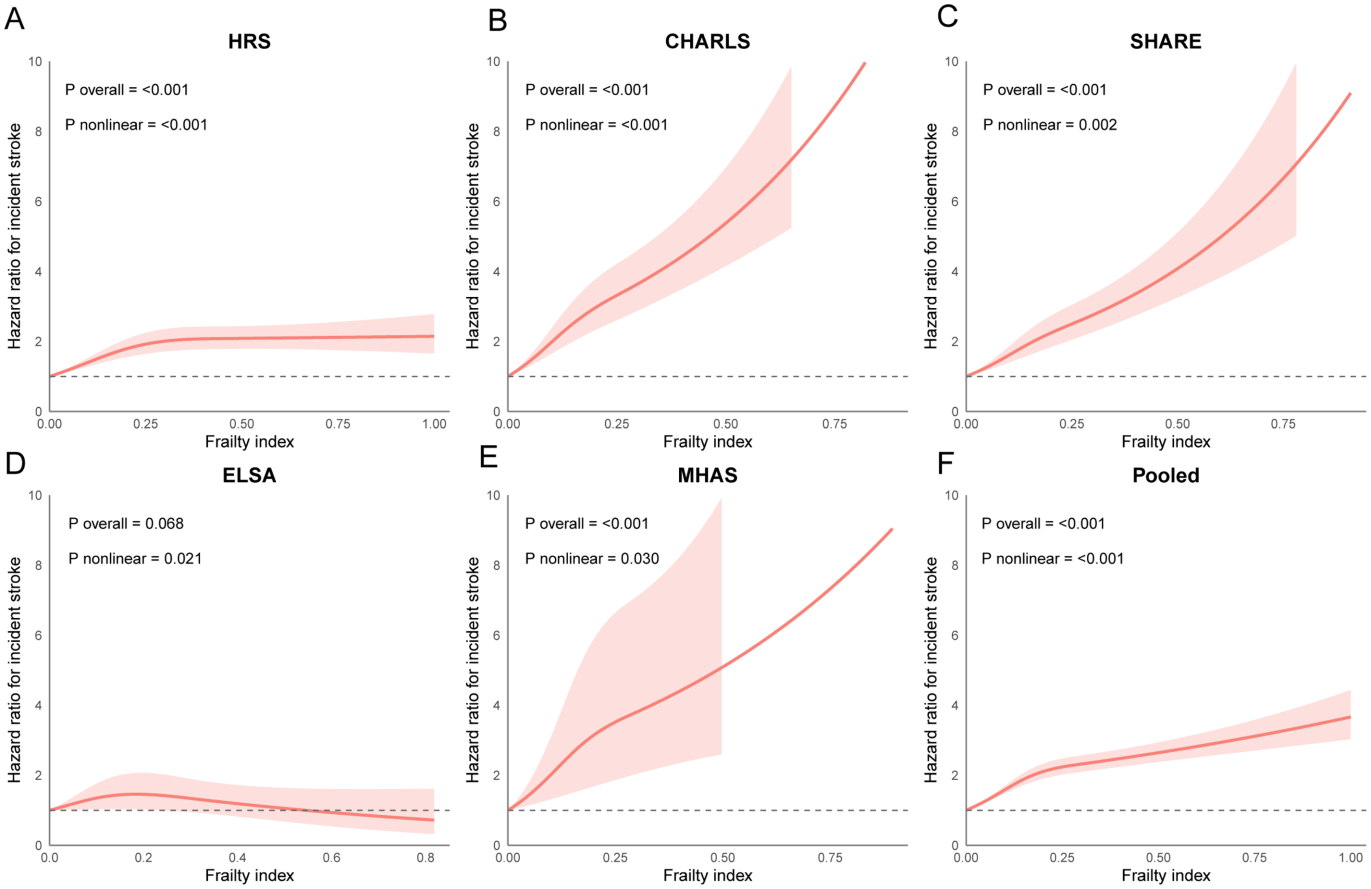
Restricted cubic spline analyses of baseline frailty index and incident stroke. **(A)** HRS; **(B)** CHARLS; **(C)** SHARE; **(D)** ELSA; **(E)** MHAS; and **(F)** pooled analysis.

RCS analyses of baseline FI (Figure 6) suggested nonlinear associations between FI and incident stroke. In the pooled analysis, stroke risk rose progressively as FI increased, with steeper increases at higher FI levels. This pattern suggests that accumulating deficits may have compounding implications for cerebrovascular vulnerability.

**Figure 7:**
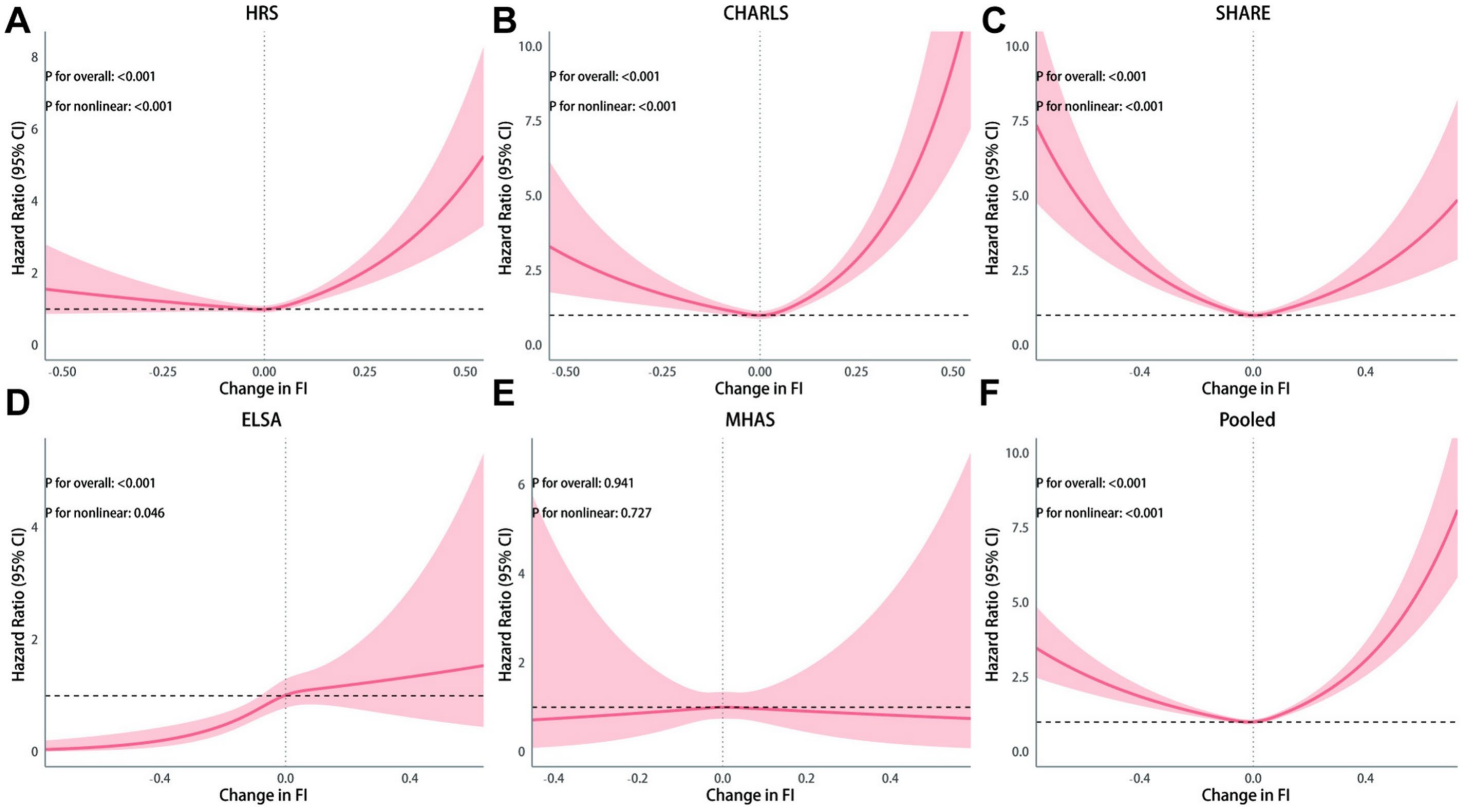
Restricted cubic spline analyses of frailty-index change and incident stroke. **(A)** HRS; **(B)** CHARLS; **(C)** SHARE; **(D)** ELSA; **(E)** MHAS; and **(F)** pooled analysis.

RCS analyses of FI change (Figure 7) showed that longitudinal increases in FI were associated with higher stroke risk in the pooled model. The pooled curve was relatively flat below the reference value of no FI change but rose as FI increased between waves, suggesting that worsening frailty vulnerability was more consistently informative than observed reductions in FI over the available follow-up intervals. Observed reductions in FI were not consistently associated with lower stroke risk, indicating that short-term improvement in frailty vulnerability did not clearly translate into measurable reduction in subsequent stroke risk in these observational data.

### 3.5 Exploratory mediation and two-wave cross-lagged analyses

**Figure 8:**
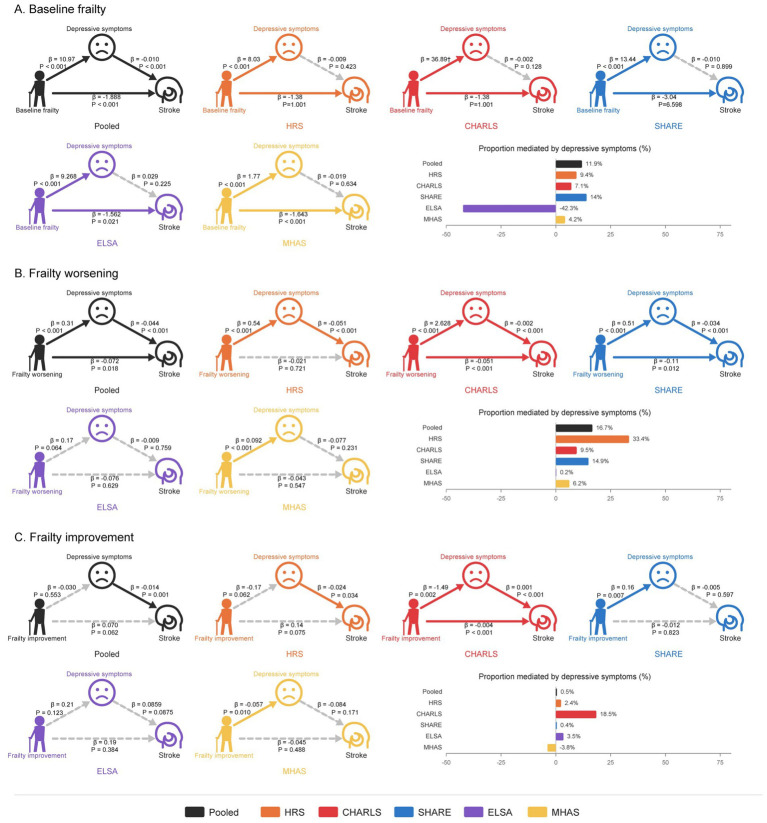
Exploratory mediation analyses evaluating depressive symptoms as a pathway marker. **(A)** baseline frailty; **(B)** frailty worsening; and **(C)** frailty improvement. Each panel presents results for the pooled analysis and the HRS, CHARLS, SHARE, ELSA, and MHAS cohorts.

Mediation-related analyses (Figure 8) evaluated depressive symptoms as a pathway marker in associations between frailty-related exposures and incident stroke. Across baseline frailty, frailty increase, and frailty improvement models, depressive symptoms statistically accounted for part of selected frailty-stroke associations, but patterns varied by cohort and exposure definition. The baseline model was interpreted as a cross-sectional statistical decomposition of the baseline frailty-stroke association, whereas the dynamic models evaluated frailty increase and frailty improvement with clearer temporal ordering. These analyses were interpreted as exploratory statistical decompositions rather than proof of causal mediation.

**Figure 9:**
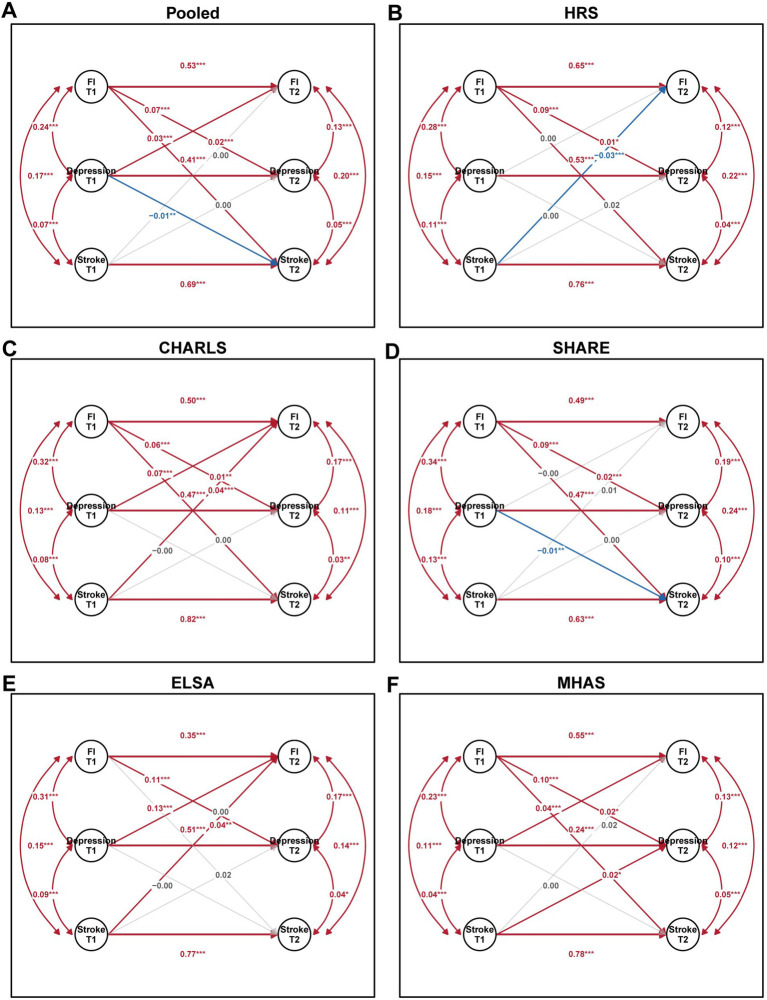
Two-wave cross-lagged panel models of elevated frailty status, depressive symptoms, and stroke status across cohorts and pooled analysis. **(A)** pooled analysis; **(B)** HRS; **(C)** CHARLS; **(D)** SHARE; **(E)** ELSA; and **(F)** MHAS.

Two-wave CLPM analyses (Figure 9) used the elevated-FI threshold of FI ≥ 0.10 and estimated autoregressive, cross-lagged, baseline-correlation, and follow-up residual-correlation paths among elevated frailty vulnerability, depressive symptoms, and stroke status. Autoregressive paths were generally strongest, indicating stability over time. CLPM paths suggested selective prospective associations from elevated frailty vulnerability to later depressive symptoms or stroke in some cohorts, but effect sizes were small and should be interpreted as exploratory temporal associations rather than causal within-person effects.

After Benjamini-Hochberg FDR correction at *q* < 0.05, the primary cohort-specific Cox findings remained robust: higher baseline FI remained associated with incident stroke in HRS, CHARLS, SHARE, and MHAS, whereas the ELSA Cox association remained non-significant. Subgroup Cox analyses were broadly unchanged after correction. RCS analyses generally supported nonlinear associations for baseline FI and FI change, although selected cohort-specific nonlinear terms were attenuated after correction. Mediation-related analyses involving baseline frailty and frailty increase remained supportive in selected cohorts, whereas frailty-improvement pathways were weaker after correction. In the two-wave CLPM, most cross-lagged paths were attenuated and did not remain significant after FDR correction. These results support the robustness of the main Cox findings while reinforcing the exploratory interpretation of secondary nonlinear, mediation-related, subgroup, and cross-lagged analyses (Supplementary Table S12).

## 4 Discussion

### 4.1 Principal findings

This harmonized multi-cohort study yielded five main findings. First, greater baseline frailty was associated with higher incident stroke risk in four of five cohorts, whereas the association was attenuated and nonsignificant in ELSA in the main Cox analysis. Second, substantial between-cohort heterogeneity indicated that cohort-specific estimates were more informative than a single common pooled effect. Third, Fine-Gray sensitivity analyses suggested that competing mortality may have contributed to, but did not fully explain, the weaker ELSA Cox finding. Fourth, nonlinear analyses indicated that accumulated frailty burden and worsening FI over time were associated with higher stroke risk, whereas observed frailty improvement did not clearly confer a corresponding reduction in risk. Fifth, depressive symptoms appeared relevant to selected frailty-stroke pathways but did not fully explain the association.

The overall pattern suggests that frailty is relevant to stroke risk across diverse aging populations, but the magnitude and statistical strength of this association differed across cohorts. Higher FI was associated with incident stroke in HRS, CHARLS, SHARE, and MHAS, whereas the association was weaker and not statistically significant in ELSA in the main Cox models. Stronger estimates in CHARLS, SHARE, and MHAS may reflect differences in population health profiles, frailty distributions, vascular risk burden, social and healthcare contexts, and follow-up structures. The attenuated Cox association in ELSA may reflect event ascertainment, follow-up duration, sample composition, competing mortality, residual confounding, or proportional-hazards behavior. The Fine-Gray sensitivity analysis indicates that competing mortality or informative censoring may be relevant, but this discrepancy should not be attributed solely to competing mortality. These cross-cohort differences support cohort-specific interpretation rather than reliance on a single pooled estimate.

### 4.2 Interpretation in relation to prior work

Prior studies have established frailty as a multidimensional geriatric syndrome and have shown that both the physical phenotype and the deficit-accumulation FI predict adverse outcomes (3–8). Recent studies focused on stroke have linked frailty to stroke prognosis, stroke incidence, frailty trajectories, and genetic evidence relating frailty to stroke risk (9, 10, 35–38). The present study extends this work by examining baseline FI, FI change, nonlinear dose–response patterns, competing mortality, and depressive symptoms across five harmonized international cohorts.

Published literature further supports subtype-specific interpretation. In an observational and genetic analysis, Renedo et al. reported that a higher Hospital Frailty Risk Score was associated with increased risk of any stroke and included subtype-specific analyses for ischemic and hemorrhagic stroke (39). Other studies reported prognostic associations of frailty within ischemic stroke (10), intracerebral hemorrhage (40), and aneurysmal subarachnoid hemorrhage (41). These findings support our decision to avoid interpreting all-cause incident stroke as a single mechanistic endpoint and highlight the need for future analyses with adjudicated stroke subtype information.

Research on depression, psychosocial stress, and stroke has demonstrated that psychological vulnerability is associated with higher stroke risk (14, 15, 42). The vascular depression hypothesis and related mechanistic work implicate small-vessel disease, inflammation, autonomic dysregulation, and vascular pathology (16–20). By integrating depressive symptoms with dynamic frailty measures, this study highlights that physical and psychological vulnerability may jointly contribute to stroke risk.

### 4.3 Potential mechanisms

Frailty may increase stroke risk through cumulative dysregulation across inflammatory, neuroendocrine, metabolic, vascular, and behavioral systems. Low-grade inflammation and vascular dysfunction may promote atherosclerosis, endothelial injury, thrombosis, and cerebral small-vessel disease (18–20, 43). Frailty may also reduce mobility and social participation, leading to depressive symptoms, inactivity, poorer diet, and reduced adherence to vascular-risk management. Social isolation and psychosocial stress may further compound depressive symptoms and cerebrovascular vulnerability (42, 44). These mechanisms provide plausible explanations for why combined frailty and mood assessment may be clinically informative, but they should not be interpreted as proof of causal mediation.

Because stroke subtype information was unavailable, the mechanisms discussed here should be interpreted as plausible pathways linking frailty with overall incident stroke rather than as subtype-specific mechanisms. Ischemic stroke, intracerebral hemorrhage, and subarachnoid hemorrhage differ in pathophysiology, risk-factor profiles, and clinical trajectories (2, 10, 39–41). Therefore, the relative contributions of ischemic and hemorrhagic pathways could not be determined, and the frailty-stroke association should not be interpreted as reflecting a single unified mechanism.

### 4.4 Strengths and limitations

A major strength of this study is the use of five large, population-based aging cohorts spanning North America, Europe, Asia, and Latin America. Harmonized FI construction allowed broadly comparable analyses across cohorts, and the analytic framework integrated primary Cox models with subgroup analyses, nonlinear modeling, FI-change analyses, competing-risk sensitivity analyses, mediation-related analyses, and two-wave CLPMs. However, the baseline pathway analysis could not establish temporal ordering because baseline frailty and depressive symptoms were measured at the same wave. Although dynamic frailty-change models had clearer temporal sequencing, all pathway analyses remained observational and may be affected by residual confounding, measurement error, and cohort-specific survey intervals. Depressive symptoms should therefore be interpreted as a potential pathway marker rather than a proven causal mediator.

Several limitations should be noted. First, incident stroke was primarily based on self-reported physician diagnosis, supplemented by proxy, exit, linked, or cohort-specific information where available. Differences in self-report accuracy, proxy reporting, survey intervals, follow-up schedules, and supplementary ascertainment sources may have contributed to between-cohort heterogeneity and could partly explain weaker findings in ELSA. Second, stroke subtype information was not consistently available across all five harmonized cohorts; therefore, ischemic stroke, intracerebral hemorrhage, and subarachnoid hemorrhage could not be analyzed separately. If frailty is more strongly associated with one subtype, all-cause stroke analyses may dilute or obscure subtype-specific associations. Conversely, combining ischemic and hemorrhagic outcomes may mask differences in pathophysiology, risk-factor profiles, and clinical mechanisms (2, 10, 39–41). Third, although FI construction was harmonized, some cohort-specific measures required simplification and may not capture all clinically relevant deficits. The FI was calculated using a consistent prorating rule when at least 80% of items were available, but we did not conduct a separate sensitivity analysis using alternative item-completeness thresholds, such as complete-item scoring or a > =90% threshold. Fourth, frailty-change exposures were defined over adjacent survey waves, and the calendar duration of the T1-to-T2 interval varied across cohorts according to cohort-specific survey schedules. Differences in interval duration may affect the comparability of frailty-change, mediation-related, and two-wave CLPM estimates across cohorts. Fifth, frailty change was analyzed as a time-fixed exposure derived from an adjacent-wave interval rather than as a repeatedly updated time-varying covariate; therefore, the analyses did not capture all subsequent within-person changes in frailty across the full follow-up period. Sixth, differences between Cox and Fine-Gray estimates should be interpreted in light of their different estimands rather than as direct evidence of model error. The Fine-Gray model addresses death as a competing event for cumulative incidence, whereas the Cox model estimates cause-specific hazards under censoring of deaths. Discordant findings, particularly in ELSA, may therefore reflect competing mortality, informative censoring, limited event counts, or other cohort-specific sources of heterogeneity. Scaled Schoenfeld residual tests also indicated potential proportional-hazards departures in CHARLS and ELSA. Cox estimates in these cohorts should therefore be interpreted as average associations over follow-up rather than as strictly time-constant hazard ratios. Seventh, depressive symptoms were measured using different scales across cohorts; within-cohort standardization improves comparability but cannot fully eliminate measurement differences. Eighth, residual confounding remains possible because biomarkers, neuroimaging markers, detailed medication use, atrial fibrillation, diet, and health-care access were not uniformly available. Ninth, mediation-related and cross-lagged analyses were exploratory and observational, and the study was not preregistered; secondary findings should therefore be considered hypothesis-generating. Tenth, although FDR correction was applied within prespecified test families, many secondary analyses were exploratory and should not be interpreted as confirmatory evidence. Finally, the finding that frailty improvement was not clearly associated with lower stroke risk should be interpreted cautiously because follow-up after improvement may have been insufficient, the FI ≥ 0.10 transition may be affected by measurement error or regression to the mean, and improvement in global frailty burden may not fully reverse established vascular pathology or cumulative cerebrovascular risk.

### 4.5 Clinical and research implications

The findings suggest that repeated frailty assessment may help identify older adults at increased stroke risk, particularly when vulnerability worsens over time. The asymmetric pattern between frailty worsening and frailty improvement suggests that increasing vulnerability may be more informative for near-term risk stratification than short-term FI improvement. However, improvement in FI should not be assumed to normalize stroke risk. Older adults whose frailty improves may still require vascular-risk assessment, mood evaluation, physical activity support, and management of hypertension, diabetes, heart disease, and other stroke risk factors (45). Because depressive symptoms may be intertwined with frailty progression, integrated physical and psychological assessment may be more informative than either domain alone. Future studies with adjudicated stroke outcomes and subtype classification are needed to determine whether frailty is differentially associated with ischemic stroke, intracerebral hemorrhage, or subarachnoid hemorrhage. Intervention studies are also needed to test whether programs targeting frailty, depressive symptoms, vascular-risk factors, or their combination can reduce incident stroke risk.

## 5 Conclusion

Higher baseline frailty burden was associated with incident stroke in most, but not all, harmonized aging cohorts, with substantial heterogeneity across populations. Nonlinear and dynamic analyses suggested that accumulated frailty burden and worsening FI over time were clinically informative, whereas observed frailty improvement did not clearly translate into reduced stroke risk in this observational analysis. Depressive symptoms appeared relevant to selected frailty-stroke pathways, supporting repeated frailty assessment and integrated mood evaluation while emphasizing the need for confirmatory studies and cautious cohort-specific interpretation.

## Data Availability

The datasets presented in this study can be found in online repositories. The names of the repository/repositories and accession number(s) can be found below: The data analyzed in this study are publicly available to registered researchers through the official repositories of each cohort. HRS data are available from the Health and Retirement Study Data Portal/RAND HRS Products; the RAND HRS Longitudinal File requires free registration and login, and no accession number is assigned. CHARLS data are available from the China Health and Retirement Longitudinal Study Data Portal; Harmonized CHARLS Version D is provided through CHARLS/Gateway to Global Aging Data, with dataset SHA1: 5CB01C2398DE1BC7D718299EE2D006D55D8D9BDF. SHARE data are available from the SHARE Research Data Center to registered users; the relevant SHARE release uses wave-specific DOIs, including Waves 1–9, for example 10.6103/SHARE.w1.900 through 10.6103/SHARE.w9.900. ELSA data are available from the UK Data Service under Study Number SN 5050, DOI 10.5255/UKDA-SN-5050-37. MHAS data are available from the Mexican Health and Aging Study Data Products repository after free registration; public-use files and documentation are available through MHASweb, and Harmonized MHAS is produced with the Gateway to Global Aging Data. Repository names and links: HRS: Health and Retirement Study Data Portal/RAND HRS Products. CHARLS: China Health and Retirement Longitudinal Study Data Portal. SHARE: SHARE Research Data Center, SHARE-ERIC. ELSA: UK Data Service, Study Number 5050. MHAS: Mexican Health and Aging Study Data Products/Harmonized MHAS.
