# Supplementary material for: Dynamic frailty and depressive symptoms in relation to incident stroke: findings from five harmonized longitudinal cohorts
Source: Front Neurol. 2026 Jul 14;17:1880619. doi: 10.3389/fneur.2026.1880619 (PMC13407644; doi:10.3389/fneur.2026.1880619)
Supplement: Supplementary file 1 [file Data_Sheet_1.docx]

**Supplementary Table S1. Cohort-specific survey schedules and analytic sample construction**

| **Cohort** | **Country / region** | **Baseline wave** | **Baseline year** | **Follow-up waves used for incident stroke** | **Approximate survey interval** | **Main Cox analytic N** | **Incident stroke events** |
| --- | --- | --- | --- | --- | --- | --- | --- |
| **HRS** | United States | Wave 5 | 2000 | Waves 6–14 | Approximately biennial | 14,586 | 1,841 |
| **CHARLS** | China | Wave 1 | 2011 | Waves 2–4 | Approximately biennial | 11,202 | 828 |
| **SHARE** | Europe/Israel | Wave 4 | 2011 | Waves 5–8 | Approximately biennial, with cohort-specific variation | 36,593 | 1,867 |
| **ELSA** | England | Wave 2 | 2004 | Waves 3–9 | Approximately biennial | 8,660 | 378 |
| **MHAS** | Mexico | Wave 1 | 2001 | Waves 2–5 | Variable multi-year intervals | 10,441 | 175 |

Note. Eligibility required participation in the cohort-specific baseline wave, no self-reported stroke at baseline, a calculable baseline frailty index (FI), and available follow-up information for incident stroke. The same 24-item FI scoring framework and the same ≥80% item-completeness rule were applied across cohorts. Cohort-specific variable mappings were used only to harmonize source items to common deficit domains rather than to change the FI scoring rule. The approximate survey intervals for each cohort include approximately biennial follow-up for HRS, CHARLS, and ELSA; approximately biennial follow-up with cohort-specific variation for SHARE; and variable multi-year intervals for MHAS. Main exclusions were prevalent baseline stroke, missing or invalid baseline FI, missing incident-stroke status or follow-up time, or missing key model covariates. The FI was calculated by prorating observed deficits when at least 80% of the 24 items were available and was set to missing when fewer than 19 items were observed. All models used model-specific complete-case analysis. Participants without incident stroke were censored at death, loss to follow-up, or the final available follow-up wave, whichever occurred first. The listed analytic N and event count refer to the main fully adjusted baseline-FI Cox analytic sample; repeated-measure, mediation, restricted cubic spline, and cross-lagged panel analyses used analysis-specific samples.

**Supplementary Table S2. Covariate definitions, timing of measurement, and rationale for adjustment**

| **Variable** | **Timing of measurement** | **Operational definition** | **Rationale for inclusion** |
| --- | --- | --- | --- |
| **Age** | Baseline wave | Continuous, years | Strong determinant of frailty accumulation and stroke risk |
| **Sex** | Baseline wave | Female or male | Associated with frailty distribution and stroke epidemiology |
| **Education** | Baseline wave | Below secondary vs secondary or above | Marker of socioeconomic position and lifelong health resources |
| **Employment** | Baseline wave | Currently employed vs not currently employed | Marker of socioeconomic status, functional capacity, and health selection |
| **Marital status** | Baseline wave | Married/cohabiting vs not partnered | Proxy for household and social support |
| **Children** | Baseline wave | One or more children vs none | Proxy for family support and household resources |
| **Smoking** | Baseline wave | Current smoker vs never/former smoker | Established vascular risk factor and related to frailty progression |
| **Drinking** | Baseline wave | Current drinker vs non-drinker | Behavioral factor associated with vascular and general health |
| **Social activity** | Baseline wave | Any harmonized social/community activity vs none | Captures social engagement, isolation, and psychosocial resources |
| **Physical activity** | Baseline wave | Active vs inactive using harmonized cohort indicators | Strongly related to frailty status, mobility, and cardiovascular risk |
| **Hypertension** | Baseline wave | Self-reported physician diagnosis, yes/no | Major vascular risk factor for incident stroke |
| **Diabetes** | Baseline wave | Self-reported physician diagnosis, yes/no | Major vascular/metabolic risk factor for stroke and frailty |
| **Heart disease** | Baseline wave | Self-reported physician diagnosis, yes/no | Captures baseline cardiovascular disease burden |
| **Depressive symptoms** | Baseline wave for main Cox models; post-frailty-change wave for mediation analyses | Cohort-specific scale score standardized within cohort; binary status by established cut-points | Psychosocial factor related to frailty and stroke risk; evaluated as mediator in exploratory mediation analyses |
| **Cohort indicator** | Pooled analyses only | HRS, CHARLS, SHARE, ELSA, or MHAS | Accounts for cohort-level differences in design, geography, measurement, and follow-up |

*Note. All covariates were measured at the cohort-specific baseline wave unless otherwise stated. Depressive symptoms were treated as a baseline covariate in the main Cox models and as a candidate mediator, measured at the post-frailty-change wave, in the exploratory mediation analyses. The cohort indicator was included in pooled analyses only.*

**Supplementary Table S3. Covariate and key-variable missingness by cohort**

| **Cohort** | **Variable** | **Total N** | **Non-missing N** | **Missing (%)** | **Unique non-missing values** |
| --- | --- | --- | --- | --- | --- |
| **HRS** | fi | 40,851 | 40,851 | 0.0 | 42 |
| **HRS** | time | 40,851 | 40,851 | 0.0 | 8 |
| **HRS** | cr_status | 40,851 | 40,851 | 0.0 | 3 |
| **HRS** | age | 40,851 | 18,024 | 55.9 | 748 |
| **HRS** | gender | 40,851 | 40,851 | 0.0 | 2 |
| **HRS** | edu | 40,851 | 9,702 | 76.3 | 5 |
| **HRS** | employ | 40,851 | 4,200 | 89.7 | 33 |
| **HRS** | marital | 40,851 | 0 | 100.0 | 0 |
| **HRS** | child | 40,851 | 12,108 | 70.4 | 12 |
| **HRS** | smoke | 40,851 | 17,843 | 56.3 | 2 |
| **HRS** | drink | 40,851 | 18,022 | 55.9 | 2 |
| **HRS** | social | 40,851 | 0 | 100.0 | 0 |
| **HRS** | physical | 40,851 | 6,701 | 83.6 | 4 |
| **CHARLS** | fi | 25,100 | 25,100 | 0.0 | 75 |
| **CHARLS** | time | 25,100 | 25,100 | 0.0 | 2 |
| **CHARLS** | cr_status | 25,100 | 25,100 | 0.0 | 3 |
| **CHARLS** | age | 25,100 | 17,055 | 32.1 | 82 |
| **CHARLS** | gender | 25,100 | 25,092 | 0.0 | 2 |
| **CHARLS** | edu | 25,100 | 25,057 | 0.2 | 10 |
| **CHARLS** | employ | 25,100 | 16,784 | 33.1 | 2 |
| **CHARLS** | marital | 25,100 | 0 | 100.0 | 0 |
| **CHARLS** | child | 25,100 | 17,222 | 31.4 | 11 |
| **CHARLS** | smoke | 25,100 | 17,073 | 32.0 | 2 |
| **CHARLS** | drink | 25,100 | 17,058 | 32.0 | 2 |
| **CHARLS** | social | 25,100 | 0 | 100.0 | 0 |
| **CHARLS** | physical | 25,100 | 0 | 100.0 | 0 |
| **SHARE** | fi | 143,367 | 143,367 | 0.0 | 81 |
| **SHARE** | time | 143,367 | 143,367 | 0.0 | 3 |
| **SHARE** | cr_status | 143,367 | 143,367 | 0.0 | 3 |
| **SHARE** | age | 143,367 | 54,797 | 61.8 | 76 |
| **SHARE** | gender | 143,367 | 143,367 | 0.0 | 2 |
| **SHARE** | edu | 143,367 | 143,367 | 0.0 | 3 |
| **SHARE** | employ | 143,367 | 54,146 | 62.2 | 2 |
| **SHARE** | marital | 143,367 | 0 | 100.0 | 0 |
| **SHARE** | child | 143,367 | 29,519 | 79.4 | 18 |
| **SHARE** | smoke | 143,367 | 53,735 | 62.5 | 2 |
| **SHARE** | drink | 143,367 | 48,674 | 66.0 | 2 |
| **SHARE** | social | 143,367 | 0 | 100.0 | 0 |
| **SHARE** | physical | 143,367 | 0 | 100.0 | 0 |
| **ELSA** | fi | 19,358 | 19,358 | 0.0 | 52 |
| **ELSA** | time | 19,358 | 19,358 | 0.0 | 6 |
| **ELSA** | cr_status | 19,358 | 19,358 | 0.0 | 3 |
| **ELSA** | age | 19,358 | 8,988 | 53.6 | 61 |
| **ELSA** | gender | 19,358 | 19,358 | 0.0 | 2 |
| **ELSA** | edu | 19,358 | 17,572 | 9.2 | 4 |
| **ELSA** | employ | 19,358 | 8,987 | 53.6 | 2 |
| **ELSA** | marital | 19,358 | 0 | 100.0 | 0 |
| **ELSA** | child | 19,358 | 8,988 | 53.6 | 12 |
| **ELSA** | smoke | 19,358 | 8,986 | 53.6 | 2 |
| **ELSA** | drink | 19,358 | 7,826 | 59.6 | 2 |
| **ELSA** | social | 19,358 | 8,669 | 55.2 | 5 |
| **ELSA** | physical | 19,358 | 3,242 | 83.3 | 4 |
| **MHAS** | fi | 26,448 | 26,448 | 0.0 | 89 |
| **MHAS** | time | 26,448 | 26,448 | 0.0 | 3 |
| **MHAS** | cr_status | 26,448 | 26,448 | 0.0 | 3 |
| **MHAS** | age | 26,448 | 14,795 | 44.1 | 84 |
| **MHAS** | gender | 26,448 | 26,448 | 0.0 | 2 |
| **MHAS** | edu | 26,448 | 26,084 | 1.4 | 3 |
| **MHAS** | employ | 26,448 | 14,705 | 44.4 | 2 |
| **MHAS** | marital | 26,448 | 0 | 100.0 | 0 |
| **MHAS** | child | 26,448 | 14,795 | 44.1 | 22 |
| **MHAS** | smoke | 26,448 | 14,783 | 44.1 | 2 |
| **MHAS** | drink | 26,448 | 14,784 | 44.1 | 2 |
| **MHAS** | social | 26,448 | 0 | 100.0 | 0 |
| **MHAS** | physical | 26,448 | 0 | 100.0 | 0 |

*Note. N_total indicates the number of participants in the cohort-specific source dataset used for this diagnostic table. N_nonmissing indicates the number with non-missing data for the corresponding variable, Missing_pct is the percentage of missing observations, and Unique_nonmissing is the number of distinct observed values. Variable names follow the harmonized analysis datasets. This table was used to evaluate model-specific complete-case availability and covariate missingness patterns.*

**Supplementary Table S4. Distributional properties and age gradients of the 24-item frailty index by cohort**

| **Cohort** | **N** | **Age, mean (SD)** | **FI, mean (SD)** | **FI, median (IQR)** | **FI range** | **FI skewness** | **Spearman ρ (age–FI)** |
| --- | --- | --- | --- | --- | --- | --- | --- |
| **HRS** | 14,421 | 65.8 (9.2) | 0.192 (0.183) | 0.167 (0.083–0.250) | 0.000–1.000 | 1.696 | 0.158 (<0.001) |
| **CHARLS** | 11,202 | 61.4 (7.9) | 0.130 (0.122) | 0.118 (0.059–0.176) | 0.000–0.882 | 1.711 | 0.201 (<0.001) |
| **SHARE** | 36,593 | 64.8 (9.2) | 0.147 (0.097) | 0.136 (0.091–0.182) | 0.000–0.909 | 1.555 | 0.200 (<0.001) |
| **ELSA** | 8,660 | 65.8 (9.8) | 0.141 (0.124) | 0.095 (0.045–0.182) | 0.000–0.818 | 1.437 | 0.249 (<0.001) |
| **MHAS** | 10,439 | 64.1 (8.8) | 0.157 (0.117) | 0.150 (0.067–0.200) | 0.000–0.895 | 1.199 | 0.229 (<0.001) |

*Note. FI, frailty index; SD, standard deviation; IQR, interquartile range. The validation sample includes participants with valid age and a calculable FI. Positive skewness indicates a right-skewed FI distribution; Spearman correlations (ρ) test the expected positive age gradient of the FI, with the associated P value shown in parentheses. These are descriptive validation checks of the harmonized FI and should not be interpreted as formal psychometric validation.*

**Supplementary Table S5. Frailty index values by age group and cohort**

| **Cohort** | **Age group, years** | **N** | **FI, mean (SD)** | **FI, median (IQR)** |
| --- | --- | --- | --- | --- |
| **HRS** | HRS | HRS | HRS | HRS |
|  | 50–59 | 4,084 | 0.162 (0.169) | 0.111 (0.056–0.222) |
|  | 60–69 | 5,727 | 0.190 (0.178) | 0.167 (0.083–0.250) |
|  | 70–79 | 3,296 | 0.209 (0.189) | 0.167 (0.083–0.278) |
|  | 80+ | 1,314 | 0.254 (0.212) | 0.167 (0.111–0.333) |
| **CHARLS** | CHARLS | CHARLS | CHARLS | CHARLS |
|  | 50–59 | 5,448 | 0.108 (0.105) | 0.059 (0.059–0.167) |
|  | 60–69 | 3,907 | 0.140 (0.124) | 0.118 (0.059–0.176) |
|  | 70–79 | 1,565 | 0.165 (0.141) | 0.118 (0.059–0.235) |
|  | 80+ | 282 | 0.221 (0.175) | 0.176 (0.111–0.333) |
| **SHARE** | SHARE | SHARE | SHARE | SHARE |
|  | 50–59 | 12,025 | 0.128 (0.083) | 0.095 (0.091–0.182) |
|  | 60–69 | 13,472 | 0.141 (0.090) | 0.136 (0.091–0.182) |
|  | 70–79 | 8,340 | 0.166 (0.103) | 0.136 (0.091–0.227) |
|  | 80+ | 2,756 | 0.203 (0.133) | 0.182 (0.095–0.273) |
| **ELSA** | ELSA | ELSA | ELSA | ELSA |
|  | 50–59 | 2,881 | 0.107 (0.107) | 0.091 (0.045–0.143) |
|  | 60–69 | 2,791 | 0.134 (0.118) | 0.091 (0.045–0.182) |
|  | 70–79 | 2,023 | 0.162 (0.124) | 0.136 (0.091–0.227) |
|  | 80+ | 965 | 0.216 (0.147) | 0.182 (0.091–0.318) |
| **MHAS** | MHAS | MHAS | MHAS | MHAS |
|  | 50–59 | 3,595 | 0.126 (0.099) | 0.100 (0.050–0.200) |
|  | 60–69 | 4,138 | 0.161 (0.114) | 0.150 (0.100–0.211) |
|  | 70–79 | 2,128 | 0.183 (0.124) | 0.150 (0.100–0.250) |
|  | 80+ | 578 | 0.228 (0.149) | 0.200 (0.105–0.300) |

*Note. FI, frailty index; SD, standard deviation; IQR, interquartile range. Age groups were defined as 50–59, 60–69, 70–79, and ≥80 years. Within every cohort the mean FI increased monotonically across successive age groups, consistent with the expected age gradient of frailty.*

**Supplementary Table S6. Fine-Gray competing-risk sensitivity analysis of baseline frailty index and incident stroke**

| **Cohort** | **Full competing- risk sample N** | **Full-sample stroke events** | **Full-sample competing deaths** | **Full-sample censored** | **M1 sHR (95% CI)** | **M2 sHR (95% CI)** | **M3 sHR (95% CI)** | **M4 sHR (95% CI)** |
| --- | --- | --- | --- | --- | --- | --- | --- | --- |
| **HRS** | 40,851 | 3,214 | 12,609 | 25,028 | 1.06 (1.03–1.10) P <0.001 | 1.41 (1.16–1.71) P <0.001 | 1.40 (1.15–1.71) P <0.001 | 1.39 (1.13–1.70) P = 0.002 |
| **CHARLS** | 25,100 | 337 | 3,062 | 21,701 | 1.50 (1.36–1.65) P <0.001 | 1.47 (1.33–1.63) P <0.001 | 1.47 (1.33–1.63) P <0.001 | 1.47 (1.33–1.63) P <0.001 |
| **SHARE** | 143,367 | 5,736 | 13,682 | 123,949 | 1.23 (1.20–1.27) P <0.001 | 1.23 (1.19–1.26) P <0.001 | 1.20 (1.13–1.28) P <0.001 | 1.24 (1.13–1.37) P <0.001 |
| **ELSA** | 19,358 | 692 | 2,480 | 16,186 | 1.11 (1.03–1.19) P = 0.005 | 1.08 (1.00–1.16) P = 0.041 | 1.08 (1.00–1.16) P = 0.049 | 1.37 (1.05–1.79) P = 0.022 |
| **MHAS** | 26,448 | 605 | 4,331 | 21,512 | 1.25 (1.17–1.35) P <0.001 | 1.24 (1.16–1.34) P <0.001 | 1.24 (1.15–1.34) P <0.001 | 1.24 (1.15–1.34) P <0.001 |
| **Pooled** | 255,124 | 10,584 | 36,164 | 208,376 | 1.22 (1.09–1.36) P <0.001 | — | — | — |

Note. sHR, subdistribution hazard ratio from Fine-Gray competing-risk regression, with death treated as the competing event; CI, confidence interval. sHRs are expressed per 0.1-unit increase in the frailty index. Adjustment sets were: M1 (recommended primary sensitivity model), frailty index + age + sex; M2, M1 + education + employment; M3, M2 + children; M4, M3 + smoking + drinking (+ physical activity and/or social activity where harmonized data were available). Models used model-specific complete-case samples, so the effective N for M1–M4 was smaller than the listed cohort N; M2–M4 should therefore be interpreted as exploratory available-covariate models. The pooled estimate is the M1 random-effects meta-analytic sHR across the five cohorts. N, stroke events, competing deaths, and censored counts correspond to the full competing-risk sample for each cohort.

**Supplementary Table S7. Reportable primary Fine-Gray sensitivity model for baseline frailty index and incident stroke**

| **Cohort** | **Full competing-risk sample N** | **Stroke events** | **Competing deaths** | **Censored** | **M1 sHR (95% CI)** | **P value** |
| --- | --- | --- | --- | --- | --- | --- |
| **HRS** | 40,851 | 3,214 | 12,609 | 25,028 | 1.06 (1.03–1.10) | <0.001 |
| **CHARLS** | 25,100 | 337 | 3,062 | 21,701 | 1.50 (1.36–1.65) | <0.001 |
| **SHARE** | 143,367 | 5,736 | 13,682 | 123,949 | 1.23 (1.20–1.27) | <0.001 |
| **ELSA** | 19,358 | 692 | 2,480 | 16,186 | 1.11 (1.03–1.19) | 0.005 |
| **MHAS** | 26,448 | 605 | 4,331 | 21,512 | 1.25 (1.17–1.35) | <0.001 |

*Note. sHR, subdistribution hazard ratio; CI, confidence interval. Death was treated as the competing event. The reportable primary Fine-Gray sensitivity model adjusted for frailty index, age, and sex. sHRs are expressed per 0.1-unit increase in the frailty index.*

**Supplementary Table S8. Fine-Gray M1 estimates with random-effects pooled summary**

| **Cohort** | **Full competing-risk sample N** | **Stroke events** | **Competing deaths** | **Censored** | **M1 sHR (95% CI)** | **P value** | **Estimate type** |
| --- | --- | --- | --- | --- | --- | --- | --- |
| **HRS** | 40,851 | 3,214 | 12,609 | 25,028 | 1.06 (1.03–1.10) | <0.001 | Cohort-specific estimate |
| **CHARLS** | 25,100 | 337 | 3,062 | 21,701 | 1.50 (1.36–1.65) | <0.001 | Cohort-specific estimate |
| **SHARE** | 143,367 | 5,736 | 13,682 | 123,949 | 1.23 (1.20–1.27) | <0.001 | Cohort-specific estimate |
| **ELSA** | 19,358 | 692 | 2,480 | 16,186 | 1.11 (1.03–1.19) | 0.005 | Cohort-specific estimate |
| **MHAS** | 26,448 | 605 | 4,331 | 21,512 | 1.25 (1.17–1.35) | <0.001 | Cohort-specific estimate |
| **Pooled** | 255,124 | 10,584 | 36,164 | 208,376 | 1.22 (1.09–1.36) | <0.001 | Random-effects pooled estimate |

*Note. sHR, subdistribution hazard ratio; CI, confidence interval. Cohort-specific estimates are from the M1 Fine-Gray model. The pooled row is a random-effects meta-analytic summary across cohorts and should be interpreted descriptively given between-cohort heterogeneity.*

**Supplementary Table S9. Full Fine-Gray competing-risk sensitivity results across sequential adjustment models**

| **Cohort** | **Full sample N** | **Stroke events** | **Competing deaths** | **Censored** | **M1 sHR (95% CI); P** | **M2 sHR (95% CI); P** | **M3 sHR (95% CI); P** | **M4 sHR (95% CI); P** |
| --- | --- | --- | --- | --- | --- | --- | --- | --- |
| **HRS** | 40,851 | 3,214 | 12,609 | 25,028 | 1.06 (1.03–1.10) P = <0.001 | 1.41 (1.16–1.71) P = <0.001 | 1.40 (1.15–1.71) P = <0.001 | 1.39 (1.13–1.70) P = 0.002 |
| **CHARLS** | 25,100 | 337 | 3,062 | 21,701 | 1.50 (1.36–1.65) P = <0.001 | 1.47 (1.33–1.63) P = <0.001 | 1.47 (1.33–1.63) P = <0.001 | 1.47 (1.33–1.63) P = <0.001 |
| **SHARE** | 143,367 | 5,736 | 13,682 | 123,949 | 1.23 (1.20–1.27) P = <0.001 | 1.23 (1.19–1.26) P = <0.001 | 1.20 (1.13–1.28) P = <0.001 | 1.24 (1.13–1.37) P = <0.001 |
| **ELSA** | 19,358 | 692 | 2,480 | 16,186 | 1.11 (1.03–1.19) P = 0.005 | 1.08 (1.00–1.16) P = 0.041 | 1.08 (1.00–1.16) P = 0.049 | 1.37 (1.05–1.79) P = 0.022 |
| **MHAS** | 26,448 | 605 | 4,331 | 21,512 | 1.25 (1.17–1.35) P = <0.001 | 1.24 (1.16–1.34) P = <0.001 | 1.24 (1.15–1.34) P = <0.001 | 1.24 (1.15–1.34) P = <0.001 |

*Note. sHR, subdistribution hazard ratio from Fine-Gray competing-risk regression; CI, confidence interval. Death was treated as the competing event. sHRs are expressed per 0.1-unit increase in the frailty index. M1 adjusted for age and sex; M2 additionally adjusted for education and employment; M3 additionally adjusted for children; M4 additionally adjusted for available behavioral and social covariates.*

**Supplementary Table S10. Fine-Gray model-specific sample sizes and covariate adjustment sets**

| **Cohort** | **Model** | **Covariates included** | **Model N** | **Stroke events** | **Death events** | **Censored** |
| --- | --- | --- | --- | --- | --- | --- |
| **HRS** | M1 | frailty + age + gender | 18,024 | 2,072 | 7,215 | 8,737 |
| **HRS** | M2 | frailty + age + gender + edu + employ | 2,053 | 159 | 301 | 1,593 |
| **HRS** | M3 | frailty + age + gender + edu + employ + child | 2,053 | 159 | 301 | 1,593 |
| **HRS** | M4 | frailty + age + gender + edu + employ + child + smoke + drink + physical | 2,040 | 159 | 297 | 1,584 |
| **CHARLS** | M1 | frailty + age + gender | 17,055 | 243 | 2,544 | 14,268 |
| **CHARLS** | M2 | frailty + age + gender + edu + employ | 16,633 | 239 | 2,491 | 13,903 |
| **CHARLS** | M3 | frailty + age + gender + edu + employ + child | 16,633 | 239 | 2,491 | 13,903 |
| **CHARLS** | M4 | frailty + age + gender + edu + employ + child + smoke + drink | 16,607 | 239 | 2,489 | 13,879 |
| **SHARE** | M1 | frailty + age + gender | 54,797 | 2,267 | 5,920 | 46,610 |
| **SHARE** | M2 | frailty + age + gender + edu + employ | 54,145 | 2,245 | 5,718 | 46,182 |
| **SHARE** | M3 | frailty + age + gender + edu + employ + child | 11,387 | 498 | 1,332 | 9,557 |
| **SHARE** | M4 | frailty + age + gender + edu + employ + child + smoke + drink | 8,405 | 347 | 803 | 7,255 |
| **ELSA** | M1 | frailty + age + gender | 8,988 | 411 | 1,356 | 7,221 |
| **ELSA** | M2 | frailty + age + gender + edu + employ | 8,220 | 375 | 1,228 | 6,617 |
| **ELSA** | M3 | frailty + age + gender + edu + employ + child | 8,220 | 375 | 1,228 | 6,617 |
| **ELSA** | M4 | frailty + age + gender + edu + employ + child + smoke + drink + social + physical | 1,937 | 61 | 14 | 1,862 |
| **MHAS** | M1 | frailty + age + gender | 14,795 | 458 | 4,168 | 10,169 |
| **MHAS** | M2 | frailty + age + gender + edu + employ | 14,687 | 457 | 4,138 | 10,092 |
| **MHAS** | M3 | frailty + age + gender + edu + employ + child | 14,687 | 457 | 4,138 | 10,092 |
| **MHAS** | M4 | frailty + age + gender + edu + employ + child + smoke + drink | 14,676 | 457 | 4,133 | 10,086 |

*Note. Model-specific N, stroke events, death events, and censored counts reflect complete-case availability for the listed covariates in each cohort-specific Fine-Gray model. Formula variables are shown as implemented in the analysis dataset.*

**Supplementary Table S11. Fine-Gray model-specific sample sizes, covariate adjustment sets, and interpretation**

| **Cohort** | **Model** | **Covariates included** | **Model N** | **Stroke events** | **Death events** | **Censored** | **Interpretation** |
| --- | --- | --- | --- | --- | --- | --- | --- |
| **HRS** | M1 | frailty + age + gender | 18,024 | 2,072 | 7,215 | 8,737 | Recommended primary Fine-Gray sensitivity model |
| **HRS** | M2 | frailty + age + gender + edu + employ | 2,053 | 159 | 301 | 1,593 | Supplementary model; interpret with model-specific N |
| **HRS** | M3 | frailty + age + gender + edu + employ + child | 2,053 | 159 | 301 | 1,593 | Exploratory available-covariate model; not fully adjusted |
| **HRS** | M4 | frailty + age + gender + edu + employ + child + smoke + drink + physical | 2,040 | 159 | 297 | 1,584 | Exploratory available-covariate model; not fully adjusted |
| **CHARLS** | M1 | frailty + age + gender | 17,055 | 243 | 2,544 | 14,268 | Recommended primary Fine-Gray sensitivity model |
| **CHARLS** | M2 | frailty + age + gender + edu + employ | 16,633 | 239 | 2,491 | 13,903 | Supplementary model; interpret with model-specific N |
| **CHARLS** | M3 | frailty + age + gender + edu + employ + child | 16,633 | 239 | 2,491 | 13,903 | Exploratory available-covariate model; not fully adjusted |
| **CHARLS** | M4 | frailty + age + gender + edu + employ + child + smoke + drink | 16,607 | 239 | 2,489 | 13,879 | Exploratory available-covariate model; not fully adjusted |
| **SHARE** | M1 | frailty + age + gender | 54,797 | 2,267 | 5,920 | 46,610 | Recommended primary Fine-Gray sensitivity model |
| **SHARE** | M2 | frailty + age + gender + edu + employ | 54,145 | 2,245 | 5,718 | 46,182 | Supplementary model; interpret with model-specific N |
| **SHARE** | M3 | frailty + age + gender + edu + employ + child | 11,387 | 498 | 1,332 | 9,557 | Exploratory available-covariate model; not fully adjusted |
| **SHARE** | M4 | frailty + age + gender + edu + employ + child + smoke + drink | 8,405 | 347 | 803 | 7,255 | Exploratory available-covariate model; not fully adjusted |
| **ELSA** | M1 | frailty + age + gender | 8,988 | 411 | 1,356 | 7,221 | Recommended primary Fine-Gray sensitivity model |
| **ELSA** | M2 | frailty + age + gender + edu + employ | 8,220 | 375 | 1,228 | 6,617 | Supplementary model; interpret with model-specific N |
| **ELSA** | M3 | frailty + age + gender + edu + employ + child | 8,220 | 375 | 1,228 | 6,617 | Exploratory available-covariate model; not fully adjusted |
| **ELSA** | M4 | frailty + age + gender + edu + employ + child + smoke + drink + social + physical | 1,937 | 61 | 14 | 1,862 | Exploratory available-covariate model; not fully adjusted |
| **MHAS** | M1 | frailty + age + gender | 14,795 | 458 | 4,168 | 10,169 | Recommended primary Fine-Gray sensitivity model |
| **MHAS** | M2 | frailty + age + gender + edu + employ | 14,687 | 457 | 4,138 | 10,092 | Supplementary model; interpret with model-specific N |
| **MHAS** | M3 | frailty + age + gender + edu + employ + child | 14,687 | 457 | 4,138 | 10,092 | Exploratory available-covariate model; not fully adjusted |
| **MHAS** | M4 | frailty + age + gender + edu + employ + child + smoke + drink | 14,676 | 457 | 4,133 | 10,086 | Exploratory available-covariate model; not fully adjusted |

*Note. This table expands Supplementary Table S10 by adding interpretation labels for each model. M1 is the recommended primary Fine-Gray sensitivity model; later models are supplementary or exploratory when covariate availability substantially reduces the analytic sample.*

**Supplementary Table S12. Nominal and FDR-adjusted P values across primary and secondary analyses**

| **Cohort** | **Comparison / pathway** | **Effect estimate / test statistic (95% CI)** | **Nominal P** | **FDR-adjusted Q** | **Significant (FDR)** |
| --- | --- | --- | --- | --- | --- |
| **Primary Cox proportional-hazards models** | Primary Cox proportional-hazards models | Primary Cox proportional-hazards models | Primary Cox proportional-hazards models | Primary Cox proportional-hazards models | Primary Cox proportional-hazards models |
| **HRS** | Model M1 | — | <0.001 | 0.001 | Yes |
| **HRS** | Model M2 | — | <0.001 | 0.001 | Yes |
| **HRS** | Model M3 | — | <0.001 | 0.001 | Yes |
| **HRS** | Model M4 | — | <0.001 | 0.001 | Yes |
| **CHARLS** | Model M1 | — | <0.001 | 0.001 | Yes |
| **CHARLS** | Model M2 | — | <0.001 | 0.001 | Yes |
| **CHARLS** | Model M3 | — | <0.001 | 0.001 | Yes |
| **CHARLS** | Model M4 | — | <0.001 | 0.001 | Yes |
| **SHARE** | Model M1 | — | <0.001 | 0.001 | Yes |
| **SHARE** | Model M2 | — | <0.001 | 0.001 | Yes |
| **SHARE** | Model M3 | — | <0.001 | 0.001 | Yes |
| **SHARE** | Model M4 | — | <0.001 | 0.001 | Yes |
| **ELSA** | Model M1 | — | 0.550 | 0.647 | No |
| **ELSA** | Model M2 | — | 0.627 | 0.685 | No |
| **ELSA** | Model M3 | — | 0.651 | 0.685 | No |
| **ELSA** | Model M4 | — | 0.827 | 0.827 | No |
| **MHAS** | Model M1 | — | <0.001 | 0.001 | Yes |
| **MHAS** | Model M2 | — | <0.001 | 0.001 | Yes |
| **MHAS** | Model M3 | — | <0.001 | 0.001 | Yes |
| **MHAS** | Model M4 | — | <0.001 | 0.001 | Yes |
| **Subgroup Cox models** | Subgroup Cox models | Subgroup Cox models | Subgroup Cox models | Subgroup Cox models | Subgroup Cox models |
| **Pooled** | Age: 50–64 | 1.28 (1.12–1.46) | <0.001 | <0.001 | Yes |
| **Pooled** | Age: 65 and older | 1.17 (1.06–1.29) | 0.002 | 0.002 | Yes |
| **Pooled** | Drinking: No | 1.19 (1.06–1.33) | 0.004 | 0.005 | Yes |
| **Pooled** | Drinking: Yes | 1.19 (1.06–1.34) | 0.003 | 0.004 | Yes |
| **Pooled** | Education: Below high school | 1.16 (1.04–1.30) | 0.009 | 0.011 | Yes |
| **Pooled** | Education: High school or above | 1.21 (1.08–1.36) | <0.001 | 0.001 | Yes |
| **Pooled** | Employment: Unemployed | 1.15 (1.03–1.29) | 0.013 | 0.018 | Yes |
| **Pooled** | Employment: Working or retired | 1.29 (1.14–1.46) | <0.001 | <0.001 | Yes |
| **Pooled** | Marital status: Married and partnered | 1.23 (1.09–1.40) | <0.001 | 0.001 | Yes |
| **Pooled** | Marital status: Unmarried and others | 1.16 (1.04–1.30) | 0.010 | 0.013 | Yes |
| **Pooled** | Physical Activity: No/Low | 1.28 (1.11–1.47) | <0.001 | <0.001 | Yes |
| **Pooled** | Physical Activity: Yes/High | 1.17 (1.06–1.30) | 0.003 | 0.003 | Yes |
| **Pooled** | Sex: Female | 1.18 (1.06–1.30) | 0.002 | 0.003 | Yes |
| **Pooled** | Sex: Male | 1.23 (1.06–1.41) | 0.005 | 0.006 | Yes |
| **Pooled** | Smoking: No | 1.20 (1.09–1.31) | <0.001 | <0.001 | Yes |
| **Pooled** | Smoking: Yes | 1.21 (1.05–1.40) | 0.010 | 0.013 | Yes |
| **HRS** | Age: 50–64 | 1.09 (1.06–1.13) | <0.001 | <0.001 | Yes |
| **HRS** | Age: 65 and older | 1.07 (1.05–1.10) | <0.001 | <0.001 | Yes |
| **HRS** | Drinking: No | 1.07 (1.05–1.10) | <0.001 | <0.001 | Yes |
| **HRS** | Drinking: Yes | 1.08 (1.04–1.11) | <0.001 | <0.001 | Yes |
| **HRS** | Education: Below high school | 1.06 (1.03–1.09) | <0.001 | <0.001 | Yes |
| **HRS** | Education: High school or above | 1.08 (1.06–1.11) | <0.001 | <0.001 | Yes |
| **HRS** | Employment: Unemployed | 1.07 (1.05–1.09) | <0.001 | <0.001 | Yes |
| **HRS** | Employment: Working or retired | 1.08 (1.04–1.13) | <0.001 | <0.001 | Yes |
| **HRS** | Marital status: Married and partnered | 1.07 (1.04–1.09) | <0.001 | <0.001 | Yes |
| **HRS** | Marital status: Unmarried and others | 1.08 (1.05–1.11) | <0.001 | <0.001 | Yes |
| **HRS** | Physical Activity: No/Low | 1.09 (1.05–1.13) | <0.001 | <0.001 | Yes |
| **HRS** | Physical Activity: Yes/High | 1.07 (1.04–1.09) | <0.001 | <0.001 | Yes |
| **HRS** | Sex: Female | 1.09 (1.06–1.12) | <0.001 | <0.001 | Yes |
| **HRS** | Sex: Male | 1.05 (1.03–1.08) | <0.001 | <0.001 | Yes |
| **HRS** | Smoking: No | 1.08 (1.06–1.10) | <0.001 | <0.001 | Yes |
| **HRS** | Smoking: Yes | 1.05 (0.99–1.10) | 0.082 | 0.099 | No |
| **CHARLS** | Age: 50–64 | 1.41 (1.33–1.49) | <0.001 | <0.001 | Yes |
| **CHARLS** | Age: 65 and older | 1.26 (1.18–1.36) | <0.001 | <0.001 | Yes |
| **CHARLS** | Drinking: No | 1.35 (1.28–1.43) | <0.001 | <0.001 | Yes |
| **CHARLS** | Drinking: Yes | 1.32 (1.22–1.43) | <0.001 | <0.001 | Yes |
| **CHARLS** | Education: Below high school | 1.27 (1.11–1.45) | <0.001 | 0.001 | Yes |
| **CHARLS** | Education: High school or above | 1.36 (1.29–1.42) | <0.001 | <0.001 | Yes |
| **CHARLS** | Employment: Unemployed | 1.34 (1.24–1.45) | <0.001 | <0.001 | Yes |
| **CHARLS** | Employment: Working or retired | 1.35 (1.28–1.43) | <0.001 | <0.001 | Yes |
| **CHARLS** | Marital status: Married and partnered | 1.36 (1.29–1.44) | <0.001 | <0.001 | Yes |
| **CHARLS** | Marital status: Unmarried and others | 1.30 (1.20–1.42) | <0.001 | <0.001 | Yes |
| **CHARLS** | Physical Activity: No/Low | 1.41 (1.24–1.60) | <0.001 | <0.001 | Yes |
| **CHARLS** | Physical Activity: Yes/High | 1.34 (1.27–1.40) | <0.001 | <0.001 | Yes |
| **CHARLS** | Sex: Female | 1.30 (1.23–1.38) | <0.001 | <0.001 | Yes |
| **CHARLS** | Sex: Male | 1.41 (1.32–1.51) | <0.001 | <0.001 | Yes |
| **CHARLS** | Smoking: No | 1.32 (1.25–1.40) | <0.001 | <0.001 | Yes |
| **CHARLS** | Smoking: Yes | 1.39 (1.29–1.50) | <0.001 | <0.001 | Yes |
| **SHARE** | Age: 50–64 | 1.46 (1.35–1.58) | <0.001 | <0.001 | Yes |
| **SHARE** | Age: 65 and older | 1.26 (1.20–1.33) | <0.001 | <0.001 | Yes |
| **SHARE** | Drinking: No | 1.24 (1.16–1.33) | <0.001 | <0.001 | Yes |
| **SHARE** | Drinking: Yes | 1.33 (1.26–1.40) | <0.001 | <0.001 | Yes |
| **SHARE** | Education: Below high school | 1.24 (1.17–1.32) | <0.001 | <0.001 | Yes |
| **SHARE** | Education: High school or above | 1.35 (1.27–1.43) | <0.001 | <0.001 | Yes |
| **SHARE** | Employment: Unemployed | 1.26 (1.20–1.31) | <0.001 | <0.001 | Yes |
| **SHARE** | Employment: Working or retired | 1.48 (1.34–1.63) | <0.001 | <0.001 | Yes |
| **SHARE** | Marital status: Married and partnered | 1.30 (1.23–1.38) | <0.001 | <0.001 | Yes |
| **SHARE** | Marital status: Unmarried and others | 1.29 (1.22–1.37) | <0.001 | <0.001 | Yes |
| **SHARE** | Physical Activity: No/Low | 1.35 (1.28–1.43) | <0.001 | <0.001 | Yes |
| **SHARE** | Physical Activity: Yes/High | 1.21 (1.14–1.30) | <0.001 | <0.001 | Yes |
| **SHARE** | Sex: Female | 1.29 (1.22–1.36) | <0.001 | <0.001 | Yes |
| **SHARE** | Sex: Male | 1.31 (1.23–1.40) | <0.001 | <0.001 | Yes |
| **SHARE** | Smoking: No | 1.27 (1.20–1.34) | <0.001 | <0.001 | Yes |
| **SHARE** | Smoking: Yes | 1.33 (1.25–1.42) | <0.001 | <0.001 | Yes |
| **ELSA** | Age: 50–64 | 1.07 (0.92–1.24) | 0.379 | 0.413 | No |
| **ELSA** | Age: 65 and older | 1.01 (0.91–1.11) | 0.893 | 0.893 | No |
| **ELSA** | Drinking: No | 0.98 (0.85–1.13) | 0.755 | 0.755 | No |
| **ELSA** | Drinking: Yes | 1.03 (0.93–1.14) | 0.530 | 0.578 | No |
| **ELSA** | Education: Below high school | 0.98 (0.86–1.11) | 0.723 | 0.723 | No |
| **ELSA** | Education: High school or above | 1.03 (0.93–1.15) | 0.557 | 0.608 | No |
| **ELSA** | Employment: Unemployed | 1.01 (0.92–1.09) | 0.888 | 0.888 | No |
| **ELSA** | Employment: Working or retired | 1.13 (0.78–1.65) | 0.515 | 0.562 | No |
| **ELSA** | Marital status: Married and partnered | 1.06 (0.94–1.19) | 0.360 | 0.393 | No |
| **ELSA** | Marital status: Unmarried and others | 0.96 (0.86–1.08) | 0.491 | 0.491 | No |
| **ELSA** | Physical Activity: No/Low | 1.06 (0.73–1.53) | 0.776 | 0.776 | No |
| **ELSA** | Physical Activity: Yes/High | 1.01 (0.93–1.10) | 0.759 | 0.776 | No |
| **ELSA** | Sex: Female | 0.99 (0.89–1.11) | 0.918 | 0.918 | No |
| **ELSA** | Sex: Male | 1.01 (0.90–1.14) | 0.824 | 0.899 | No |
| **ELSA** | Smoking: No | 1.05 (0.91–1.20) | 0.511 | 0.558 | No |
| **ELSA** | Smoking: Yes | 0.99 (0.89–1.10) | 0.858 | 0.858 | No |
| **MHAS** | Age: 50–64 | 1.42 (1.19–1.69) | <0.001 | <0.001 | Yes |
| **MHAS** | Age: 65 and older | 1.29 (1.12–1.49) | <0.001 | <0.001 | Yes |
| **MHAS** | Drinking: No | 1.31 (1.16–1.48) | <0.001 | <0.001 | Yes |
| **MHAS** | Drinking: Yes | 1.28 (0.95–1.73) | 0.100 | 0.120 | No |
| **MHAS** | Education: Below high school | 1.36 (1.14–1.63) | <0.001 | 0.001 | Yes |
| **MHAS** | Education: High school or above | 1.28 (1.10–1.48) | <0.001 | 0.001 | Yes |
| **MHAS** | Employment: Unemployed | 1.09 (0.85–1.41) | 0.486 | 0.562 | No |
| **MHAS** | Employment: Working or retired | 1.37 (1.20–1.55) | <0.001 | <0.001 | Yes |
| **MHAS** | Marital status: Married and partnered | 1.46 (1.24–1.71) | <0.001 | <0.001 | Yes |
| **MHAS** | Marital status: Unmarried and others | 1.19 (1.02–1.39) | 0.029 | 0.035 | Yes |
| **MHAS** | Physical Activity: No/Low | 1.56 (1.17–2.08) | 0.002 | 0.003 | Yes |
| **MHAS** | Physical Activity: Yes/High | 1.27 (1.12–1.44) | <0.001 | <0.001 | Yes |
| **MHAS** | Sex: Female | 1.23 (1.07–1.42) | 0.005 | 0.006 | Yes |
| **MHAS** | Sex: Male | 1.43 (1.20–1.70) | <0.001 | <0.001 | Yes |
| **MHAS** | Smoking: No | 1.28 (1.12–1.47) | <0.001 | <0.001 | Yes |
| **MHAS** | Smoking: Yes | 1.38 (1.13–1.68) | 0.001 | 0.002 | Yes |
| **Mediation — baseline frailty (2A)** | Mediation — baseline frailty (2A) | Mediation — baseline frailty (2A) | Mediation — baseline frailty (2A) | Mediation — baseline frailty (2A) | Mediation — baseline frailty (2A) |
| **Pooled** | Indirect (mediated) effect | — | <0.001 | <0.001 | Yes |
| **Pooled** | a path (frailty → depression) | — | <0.001 | <0.001 | Yes |
| **Pooled** | b path (depression → stroke) | — | <0.001 | <0.001 | Yes |
| **Pooled** | c′ path (direct frailty → stroke) | — | <0.001 | <0.001 | Yes |
| **HRS** | Indirect (mediated) effect | — | 0.406 | 0.462 | No |
| **HRS** | a path (frailty → depression) | — | <0.001 | <0.001 | Yes |
| **HRS** | b path (depression → stroke) | — | 0.423 | 0.462 | No |
| **HRS** | c′ path (direct frailty → stroke) | — | <0.001 | <0.001 | Yes |
| **CHARLS** | Indirect (mediated) effect | — | 0.144 | 0.192 | No |
| **CHARLS** | a path (frailty → depression) | — | <0.001 | <0.001 | Yes |
| **CHARLS** | b path (depression → stroke) | — | 0.128 | 0.181 | No |
| **CHARLS** | c′ path (direct frailty → stroke) | — | <0.001 | <0.001 | Yes |
| **SHARE** | Indirect (mediated) effect | — | 0.084 | 0.134 | No |
| **SHARE** | a path (frailty → depression) | — | <0.001 | <0.001 | Yes |
| **SHARE** | b path (depression → stroke) | — | 0.090 | 0.135 | No |
| **SHARE** | c′ path (direct frailty → stroke) | — | <0.001 | <0.001 | Yes |
| **ELSA** | Indirect (mediated) effect | — | 0.214 | 0.270 | No |
| **ELSA** | a path (frailty → depression) | — | <0.001 | <0.001 | Yes |
| **ELSA** | b path (depression → stroke) | — | 0.225 | 0.270 | No |
| **ELSA** | c′ path (direct frailty → stroke) | — | 0.021 | 0.037 | Yes |
| **MHAS** | Indirect (mediated) effect | — | 0.652 | 0.652 | No |
| **MHAS** | a path (frailty → depression) | — | <0.001 | <0.001 | Yes |
| **MHAS** | b path (depression → stroke) | — | 0.634 | 0.652 | No |
| **MHAS** | c′ path (direct frailty → stroke) | — | <0.001 | <0.001 | Yes |
| **Mediation — frailty increase (2B)** | Mediation — frailty increase (2B) | Mediation — frailty increase (2B) | Mediation — frailty increase (2B) | Mediation — frailty increase (2B) | Mediation — frailty increase (2B) |
| **Pooled** | Indirect (mediated) effect | — | <0.001 | <0.001 | Yes |
| **Pooled** | a path (frailty → depression) | — | <0.001 | <0.001 | Yes |
| **Pooled** | b path (depression → stroke) | — | <0.001 | <0.001 | Yes |
| **Pooled** | c′ path (direct frailty → stroke) | — | 0.018 | 0.026 | Yes |
| **HRS** | Indirect (mediated) effect | — | <0.001 | <0.001 | Yes |
| **HRS** | a path (frailty → depression) | — | <0.001 | <0.001 | Yes |
| **HRS** | b path (depression → stroke) | — | <0.001 | <0.001 | Yes |
| **HRS** | c′ path (direct frailty → stroke) | — | 0.721 | 0.760 | No |
| **CHARLS** | Indirect (mediated) effect | — | <0.001 | <0.001 | Yes |
| **CHARLS** | a path (frailty → depression) | — | <0.001 | <0.001 | Yes |
| **CHARLS** | b path (depression → stroke) | — | <0.001 | <0.001 | Yes |
| **CHARLS** | c′ path (direct frailty → stroke) | — | <0.001 | <0.001 | Yes |
| **SHARE** | Indirect (mediated) effect | — | <0.001 | <0.001 | Yes |
| **SHARE** | a path (frailty → depression) | — | <0.001 | <0.001 | Yes |
| **SHARE** | b path (depression → stroke) | — | <0.001 | <0.001 | Yes |
| **SHARE** | c′ path (direct frailty → stroke) | — | 0.012 | 0.020 | Yes |
| **ELSA** | Indirect (mediated) effect | — | 0.754 | 0.760 | No |
| **ELSA** | a path (frailty → depression) | — | 0.064 | 0.090 | No |
| **ELSA** | b path (depression → stroke) | — | 0.760 | 0.760 | No |
| **ELSA** | c′ path (direct frailty → stroke) | — | 0.629 | 0.719 | No |
| **MHAS** | Indirect (mediated) effect | — | 0.220 | 0.292 | No |
| **MHAS** | a path (frailty → depression) | — | <0.001 | <0.001 | Yes |
| **MHAS** | b path (depression → stroke) | — | 0.231 | 0.292 | No |
| **MHAS** | c′ path (direct frailty → stroke) | — | 0.547 | 0.656 | No |
| **Mediation — frailty improvement (2C)** | Mediation — frailty improvement (2C) | Mediation — frailty improvement (2C) | Mediation — frailty improvement (2C) | Mediation — frailty improvement (2C) | Mediation — frailty improvement (2C) |
| **Pooled** | Indirect (mediated) effect | — | 0.536 | 0.633 | No |
| **Pooled** | a path (frailty → depression) | — | 0.554 | 0.633 | No |
| **Pooled** | b path (depression → stroke) | — | 0.001 | 0.009 | Yes |
| **Pooled** | c′ path (direct frailty → stroke) | — | 0.062 | 0.150 | No |
| **HRS** | Indirect (mediated) effect | — | 0.090 | 0.166 | No |
| **HRS** | a path (frailty → depression) | — | 0.062 | 0.150 | No |
| **HRS** | b path (depression → stroke) | — | 0.034 | 0.102 | No |
| **HRS** | c′ path (direct frailty → stroke) | — | 0.075 | 0.164 | No |
| **CHARLS** | Indirect (mediated) effect | — | 0.002 | 0.011 | Yes |
| **CHARLS** | a path (frailty → depression) | — | 0.002 | 0.011 | Yes |
| **CHARLS** | b path (depression → stroke) | — | <0.001 | <0.001 | Yes |
| **CHARLS** | c′ path (direct frailty → stroke) | — | <0.001 | <0.001 | Yes |
| **SHARE** | Indirect (mediated) effect | — | 0.632 | 0.659 | No |
| **SHARE** | a path (frailty → depression) | — | 0.007 | 0.028 | Yes |
| **SHARE** | b path (depression → stroke) | — | 0.597 | 0.652 | No |
| **SHARE** | c′ path (direct frailty → stroke) | — | 0.823 | 0.823 | No |
| **ELSA** | Indirect (mediated) effect | — | 0.204 | 0.288 | No |
| **ELSA** | a path (frailty → depression) | — | 0.123 | 0.210 | No |
| **ELSA** | b path (depression → stroke) | — | 0.088 | 0.166 | No |
| **ELSA** | c′ path (direct frailty → stroke) | — | 0.394 | 0.526 | No |
| **MHAS** | Indirect (mediated) effect | — | 0.188 | 0.282 | No |
| **MHAS** | a path (frailty → depression) | — | 0.010 | 0.034 | Yes |
| **MHAS** | b path (depression → stroke) | — | 0.171 | 0.274 | No |
| **MHAS** | c′ path (direct frailty → stroke) | — | 0.498 | 0.629 | No |
| **Two-wave cross-lagged panel model** | Two-wave cross-lagged panel model | Two-wave cross-lagged panel model | Two-wave cross-lagged panel model | Two-wave cross-lagged panel model | Two-wave cross-lagged panel model |
| **Pooled** | T1_Dep ~~ T1_Stroke | 0.07 (0.07 to 0.08) | <0.001 | <0.001 | Yes |
| **Pooled** | T1_FI ~~ T1_Dep | 0.24 (0.24 to 0.25) | <0.001 | <0.001 | Yes |
| **Pooled** | T1_FI ~~ T1_Stroke | 0.17 (0.16 to 0.17) | <0.001 | <0.001 | Yes |
| **Pooled** | T2_Dep ~ T1_Dep | 0.41 (0.40 to 0.42) | <0.001 | <0.001 | Yes |
| **Pooled** | T2_Dep ~ T1_FI | 0.07 (0.06 to 0.07) | <0.001 | <0.001 | Yes |
| **Pooled** | T2_Dep ~ T1_Stroke | 0.00 (-0.00 to 0.01) | 0.225 | 0.276 | No |
| **Pooled** | T2_Dep ~~ T2_Stroke | 0.05 (0.04 to 0.06) | <0.001 | <0.001 | Yes |
| **Pooled** | T2_FI ~ T1_Dep | 0.03 (0.02 to 0.03) | <0.001 | <0.001 | Yes |
| **Pooled** | T2_FI ~ T1_FI | 0.53 (0.53 to 0.54) | <0.001 | <0.001 | Yes |
| **Pooled** | T2_FI ~ T1_Stroke | 0.00 (-0.00 to 0.01) | 0.390 | 0.468 | No |
| **Pooled** | T2_FI ~~ T2_Dep | 0.13 (0.12 to 0.14) | <0.001 | <0.001 | Yes |
| **Pooled** | T2_FI ~~ T2_Stroke | 0.20 (0.20 to 0.21) | <0.001 | <0.001 | Yes |
| **Pooled** | T2_Stroke ~ T1_Dep | -0.01 (-0.01 to -0.00) | 0.002 | 0.003 | Yes |
| **Pooled** | T2_Stroke ~ T1_FI | 0.02 (0.01 to 0.02) | <0.001 | <0.001 | Yes |
| **Pooled** | T2_Stroke ~ T1_Stroke | 0.69 (0.68 to 0.70) | <0.001 | <0.001 | Yes |
| **HRS** | T1_Dep ~~ T1_Stroke | 0.11 (0.09 to 0.13) | <0.001 | <0.001 | Yes |
| **HRS** | T1_FI ~~ T1_Dep | 0.28 (0.26 to 0.29) | <0.001 | <0.001 | Yes |
| **HRS** | T1_FI ~~ T1_Stroke | 0.15 (0.14 to 0.16) | <0.001 | <0.001 | Yes |
| **HRS** | T2_Dep ~ T1_Dep | 0.53 (0.52 to 0.55) | <0.001 | <0.001 | Yes |
| **HRS** | T2_Dep ~ T1_FI | 0.09 (0.07 to 0.10) | <0.001 | <0.001 | Yes |
| **HRS** | T2_Dep ~ T1_Stroke | 0.02 (-0.00 to 0.03) | 0.053 | 0.072 | No |
| **HRS** | T2_Dep ~~ T2_Stroke | 0.04 (0.02 to 0.07) | <0.001 | <0.001 | Yes |
| **HRS** | T2_FI ~ T1_Dep | 0.00 (-0.01 to 0.02) | 0.520 | 0.598 | No |
| **HRS** | T2_FI ~ T1_FI | 0.65 (0.64 to 0.66) | <0.001 | <0.001 | Yes |
| **HRS** | T2_FI ~ T1_Stroke | -0.03 (-0.04 to -0.02) | <0.001 | <0.001 | Yes |
| **HRS** | T2_FI ~~ T2_Dep | 0.12 (0.10 to 0.14) | <0.001 | <0.001 | Yes |
| **HRS** | T2_FI ~~ T2_Stroke | 0.22 (0.20 to 0.24) | <0.001 | <0.001 | Yes |
| **HRS** | T2_Stroke ~ T1_Dep | 0.00 (-0.01 to 0.01) | 0.787 | 0.850 | No |
| **HRS** | T2_Stroke ~ T1_FI | 0.01 (0.00 to 0.02) | 0.009 | 0.013 | Yes |
| **HRS** | T2_Stroke ~ T1_Stroke | 0.76 (0.74 to 0.78) | <0.001 | <0.001 | Yes |
| **CHARLS** | T1_Dep ~~ T1_Stroke | 0.08 (0.06 to 0.09) | <0.001 | <0.001 | Yes |
| **CHARLS** | T1_FI ~~ T1_Dep | 0.32 (0.31 to 0.34) | <0.001 | <0.001 | Yes |
| **CHARLS** | T1_FI ~~ T1_Stroke | 0.13 (0.12 to 0.15) | <0.001 | <0.001 | Yes |
| **CHARLS** | T2_Dep ~ T1_Dep | 0.47 (0.45 to 0.49) | <0.001 | <0.001 | Yes |
| **CHARLS** | T2_Dep ~ T1_FI | 0.06 (0.05 to 0.08) | <0.001 | <0.001 | Yes |
| **CHARLS** | T2_Dep ~ T1_Stroke | 0.00 (-0.01 to 0.02) | 0.728 | 0.803 | No |
| **CHARLS** | T2_Dep ~~ T2_Stroke | 0.03 (0.01 to 0.05) | 0.005 | 0.005 | Yes |
| **CHARLS** | T2_FI ~ T1_Dep | 0.07 (0.06 to 0.09) | <0.001 | <0.001 | Yes |
| **CHARLS** | T2_FI ~ T1_FI | 0.50 (0.49 to 0.52) | <0.001 | <0.001 | Yes |
| **CHARLS** | T2_FI ~ T1_Stroke | 0.04 (0.03 to 0.06) | <0.001 | <0.001 | Yes |
| **CHARLS** | T2_FI ~~ T2_Dep | 0.17 (0.15 to 0.19) | <0.001 | <0.001 | Yes |
| **CHARLS** | T2_FI ~~ T2_Stroke | 0.11 (0.09 to 0.13) | <0.001 | <0.001 | Yes |
| **CHARLS** | T2_Stroke ~ T1_Dep | -0.00 (-0.01 to 0.01) | 0.421 | 0.494 | No |
| **CHARLS** | T2_Stroke ~ T1_FI | 0.01 (0.00 to 0.02) | 0.005 | 0.008 | Yes |
| **CHARLS** | T2_Stroke ~ T1_Stroke | 0.82 (0.79 to 0.85) | <0.001 | <0.001 | Yes |
| **SHARE** | T1_Dep ~~ T1_Stroke | 0.13 (0.12 to 0.14) | <0.001 | <0.001 | Yes |
| **SHARE** | T1_FI ~~ T1_Dep | 0.34 (0.33 to 0.35) | <0.001 | <0.001 | Yes |
| **SHARE** | T1_FI ~~ T1_Stroke | 0.18 (0.17 to 0.19) | <0.001 | <0.001 | Yes |
| **SHARE** | T2_Dep ~ T1_Dep | 0.47 (0.46 to 0.48) | <0.001 | <0.001 | Yes |
| **SHARE** | T2_Dep ~ T1_FI | 0.09 (0.08 to 0.10) | <0.001 | <0.001 | Yes |
| **SHARE** | T2_Dep ~ T1_Stroke | 0.00 (-0.01 to 0.01) | 0.985 | 0.985 | No |
| **SHARE** | T2_Dep ~~ T2_Stroke | 0.10 (0.09 to 0.11) | <0.001 | <0.001 | Yes |
| **SHARE** | T2_FI ~ T1_Dep | -0.00 (-0.01 to 0.01) | 0.842 | 0.885 | No |
| **SHARE** | T2_FI ~ T1_FI | 0.49 (0.48 to 0.50) | <0.001 | <0.001 | Yes |
| **SHARE** | T2_FI ~ T1_Stroke | 0.01 (-0.00 to 0.02) | 0.100 | 0.128 | No |
| **SHARE** | T2_FI ~~ T2_Dep | 0.19 (0.18 to 0.20) | <0.001 | <0.001 | Yes |
| **SHARE** | T2_FI ~~ T2_Stroke | 0.24 (0.23 to 0.25) | <0.001 | <0.001 | Yes |
| **SHARE** | T2_Stroke ~ T1_Dep | -0.01 (-0.02 to -0.00) | 0.004 | 0.006 | Yes |
| **SHARE** | T2_Stroke ~ T1_FI | 0.02 (0.01 to 0.03) | <0.001 | <0.001 | Yes |
| **SHARE** | T2_Stroke ~ T1_Stroke | 0.63 (0.61 to 0.64) | <0.001 | <0.001 | Yes |
| **ELSA** | T1_Dep ~~ T1_Stroke | 0.09 (0.07 to 0.12) | <0.001 | <0.001 | Yes |
| **ELSA** | T1_FI ~~ T1_Dep | 0.31 (0.28 to 0.33) | <0.001 | <0.001 | Yes |
| **ELSA** | T1_FI ~~ T1_Stroke | 0.15 (0.13 to 0.18) | <0.001 | <0.001 | Yes |
| **ELSA** | T2_Dep ~ T1_Dep | 0.51 (0.48 to 0.53) | <0.001 | <0.001 | Yes |
| **ELSA** | T2_Dep ~ T1_FI | 0.11 (0.09 to 0.13) | <0.001 | <0.001 | Yes |
| **ELSA** | T2_Dep ~ T1_Stroke | 0.02 (-0.01 to 0.04) | 0.192 | 0.241 | No |
| **ELSA** | T2_Dep ~~ T2_Stroke | 0.04 (0.01 to 0.08) | 0.021 | 0.021 | Yes |
| **ELSA** | T2_FI ~ T1_Dep | 0.13 (0.10 to 0.15) | <0.001 | <0.001 | Yes |
| **ELSA** | T2_FI ~ T1_FI | 0.35 (0.32 to 0.38) | <0.001 | <0.001 | Yes |
| **ELSA** | T2_FI ~ T1_Stroke | 0.04 (0.02 to 0.07) | 0.001 | 0.002 | Yes |
| **ELSA** | T2_FI ~~ T2_Dep | 0.17 (0.14 to 0.19) | <0.001 | <0.001 | Yes |
| **ELSA** | T2_FI ~~ T2_Stroke | 0.14 (0.11 to 0.16) | <0.001 | <0.001 | Yes |
| **ELSA** | T2_Stroke ~ T1_Dep | -0.00 (-0.02 to 0.01) | 0.852 | 0.885 | No |
| **ELSA** | T2_Stroke ~ T1_FI | 0.00 (-0.01 to 0.02) | 0.600 | 0.675 | No |
| **ELSA** | T2_Stroke ~ T1_Stroke | 0.77 (0.73 to 0.80) | <0.001 | <0.001 | Yes |
| **MHAS** | T1_Dep ~~ T1_Stroke | 0.04 (0.02 to 0.06) | <0.001 | <0.001 | Yes |
| **MHAS** | T1_FI ~~ T1_Dep | 0.23 (0.21 to 0.24) | <0.001 | <0.001 | Yes |
| **MHAS** | T1_FI ~~ T1_Stroke | 0.11 (0.09 to 0.13) | <0.001 | <0.001 | Yes |
| **MHAS** | T2_Dep ~ T1_Dep | 0.24 (0.22 to 0.26) | <0.001 | <0.001 | Yes |
| **MHAS** | T2_Dep ~ T1_FI | 0.10 (0.08 to 0.12) | <0.001 | <0.001 | Yes |
| **MHAS** | T2_Dep ~ T1_Stroke | 0.02 (0.00 to 0.04) | 0.038 | 0.053 | No |
| **MHAS** | T2_Dep ~~ T2_Stroke | 0.05 (0.03 to 0.07) | <0.001 | <0.001 | Yes |
| **MHAS** | T2_FI ~ T1_Dep | 0.04 (0.03 to 0.06) | <0.001 | <0.001 | Yes |
| **MHAS** | T2_FI ~ T1_FI | 0.55 (0.53 to 0.56) | <0.001 | <0.001 | Yes |
| **MHAS** | T2_FI ~ T1_Stroke | 0.02 (-0.00 to 0.03) | 0.065 | 0.086 | No |
| **MHAS** | T2_FI ~~ T2_Dep | 0.13 (0.11 to 0.15) | <0.001 | <0.001 | Yes |
| **MHAS** | T2_FI ~~ T2_Stroke | 0.12 (0.10 to 0.13) | <0.001 | <0.001 | Yes |
| **MHAS** | T2_Stroke ~ T1_Dep | 0.00 (-0.01 to 0.01) | 0.960 | 0.978 | No |
| **MHAS** | T2_Stroke ~ T1_FI | 0.02 (0.00 to 0.03) | 0.019 | 0.027 | Yes |
| **MHAS** | T2_Stroke ~ T1_Stroke | 0.78 (0.75 to 0.81) | <0.001 | <0.001 | Yes |
| **Restricted cubic spline — FI change** | Restricted cubic spline — FI change | Restricted cubic spline — FI change | Restricted cubic spline — FI change | Restricted cubic spline — FI change | Restricted cubic spline — FI change |
| **Pooled** | Nonlinear component | — | <0.001 | <0.001 | Yes |
| **Pooled** | Overall association | — | <0.001 | <0.001 | Yes |
| **HRS** | Nonlinear component | — | <0.001 | <0.001 | Yes |
| **HRS** | Overall association | — | <0.001 | <0.001 | Yes |
| **CHARLS** | Nonlinear component | — | <0.001 | <0.001 | Yes |
| **CHARLS** | Overall association | — | <0.001 | <0.001 | Yes |
| **SHARE** | Nonlinear component | — | <0.001 | <0.001 | Yes |
| **SHARE** | Overall association | — | <0.001 | <0.001 | Yes |
| **ELSA** | Nonlinear component | — | 0.046 | 0.055 | No |
| **ELSA** | Overall association | — | <0.001 | <0.001 | Yes |
| **MHAS** | Nonlinear component | — | 0.727 | 0.793 | No |
| **MHAS** | Overall association | — | 0.941 | 0.941 | No |
| **Proportional-hazards diagnostic** | Proportional-hazards diagnostic | Proportional-hazards diagnostic | Proportional-hazards diagnostic | Proportional-hazards diagnostic | Proportional-hazards diagnostic |
| **HRS** | M1 — Frailty | — | 0.553 | 0.802 | No |
| **HRS** | M1 — Global | — | 0.232 | 0.546 | No |
| **HRS** | M2 — Frailty | — | 0.646 | 0.807 | No |
| **HRS** | M2 — Global | — | 0.354 | 0.748 | No |
| **HRS** | M3 — Frailty | — | 0.640 | 0.807 | No |
| **HRS** | M3 — Global | — | 0.413 | 0.748 | No |
| **HRS** | M4 — Frailty | χ²=0.22; df=1 | 0.637 | 0.807 | No |
| **HRS** | M4 — Global | — | 0.675 | 0.819 | No |
| **CHARLS** | M1 — Frailty | — | 0.028 | 0.086 | No |
| **CHARLS** | M1 — Global | — | <0.001 | 0.002 | Yes |
| **CHARLS** | M2 — Frailty | — | 0.034 | 0.086 | No |
| **CHARLS** | M2 — Global | — | <0.001 | 0.004 | Yes |
| **CHARLS** | M3 — Frailty | — | 0.034 | 0.086 | No |
| **CHARLS** | M3 — Global | — | <0.001 | <0.001 | Yes |
| **CHARLS** | M4 — Frailty | χ²=4.55; df=1 | 0.033 | 0.086 | No |
| **CHARLS** | M4 — Global | — | <0.001 | 0.002 | Yes |
| **SHARE** | M1 — Frailty | — | 0.885 | 0.950 | No |
| **SHARE** | M1 — Global | — | 0.472 | 0.775 | No |
| **SHARE** | M2 — Frailty | — | 0.933 | 0.950 | No |
| **SHARE** | M2 — Global | — | 0.632 | 0.807 | No |
| **SHARE** | M3 — Frailty | — | 0.950 | 0.950 | No |
| **SHARE** | M3 — Global | — | 0.561 | 0.802 | No |
| **SHARE** | M4 — Frailty | χ²=0.01; df=1 | 0.939 | 0.950 | No |
| **SHARE** | M4 — Global | — | 0.504 | 0.775 | No |
| **ELSA** | M1 — Frailty | — | 0.027 | 0.086 | No |
| **ELSA** | M1 — Global | — | <0.001 | <0.001 | Yes |
| **ELSA** | M2 — Frailty | — | 0.027 | 0.086 | No |
| **ELSA** | M2 — Global | — | <0.001 | 0.002 | Yes |
| **ELSA** | M3 — Frailty | — | 0.027 | 0.086 | No |
| **ELSA** | M3 — Global | — | <0.001 | <0.001 | Yes |
| **ELSA** | M4 — Frailty | χ²=4.89; df=1 | 0.027 | 0.086 | No |
| **ELSA** | M4 — Global | — | <0.001 | <0.001 | Yes |
| **MHAS** | M1 — Frailty | — | 0.424 | 0.748 | No |
| **MHAS** | M1 — Global | — | 0.501 | 0.775 | No |
| **MHAS** | M2 — Frailty | — | 0.427 | 0.748 | No |
| **MHAS** | M2 — Global | — | 0.730 | 0.834 | No |
| **MHAS** | M3 — Frailty | — | 0.430 | 0.748 | No |
| **MHAS** | M3 — Global | — | 0.852 | 0.947 | No |
| **MHAS** | M4 — Frailty | χ²=0.65; df=1 | 0.422 | 0.748 | No |
| **MHAS** | M4 — Global | — | 0.714 | 0.834 | No |

Note. For proportional-hazards diagnostic rows, nominal P values were obtained from scaled Schoenfeld residual tests. The χ² statistics shown for the M4 frailty-index rows are the test statistics for the frailty index term in the fully adjusted cohort-specific Cox models reported in the main manuscript; df=1 for the frailty index term. Nominal P values were adjusted using the Benjamini-Hochberg false-discovery-rate procedure. Global tests are reported by nominal P and FDR-adjusted Q values because their degrees of freedom vary according to the model-specific covariate structure.

*Note. P values are nominal; Q values are Benjamini–Hochberg false-discovery-rate (FDR)–adjusted P values. “Significant (FDR)” denotes Q < 0.05. Effect estimates are hazard ratios (subgroup Cox models) or standardized path coefficients (cross-lagged panel model); an em dash (—) indicates analyses for which a single scalar effect estimate is not reported in this table (omnibus or diagnostic tests). Cox adjustment sets M1–M4 are defined in Supplementary Table S6. Mediation pathways follow the a / b / c′ / indirect-effect convention. For the proportional-hazards diagnostic, larger P values indicate no evidence against the proportional-hazards assumption.*
