# Supplementary figures and images for: Dynamic frailty and depressive symptoms in relation to incident stroke: findings from five harmonized longitudinal cohorts
Source: Front Neurol. 2026 Jul 14;17:1880619. doi: 10.3389/fneur.2026.1880619 (PMC13407644; doi:10.3389/fneur.2026.1880619)

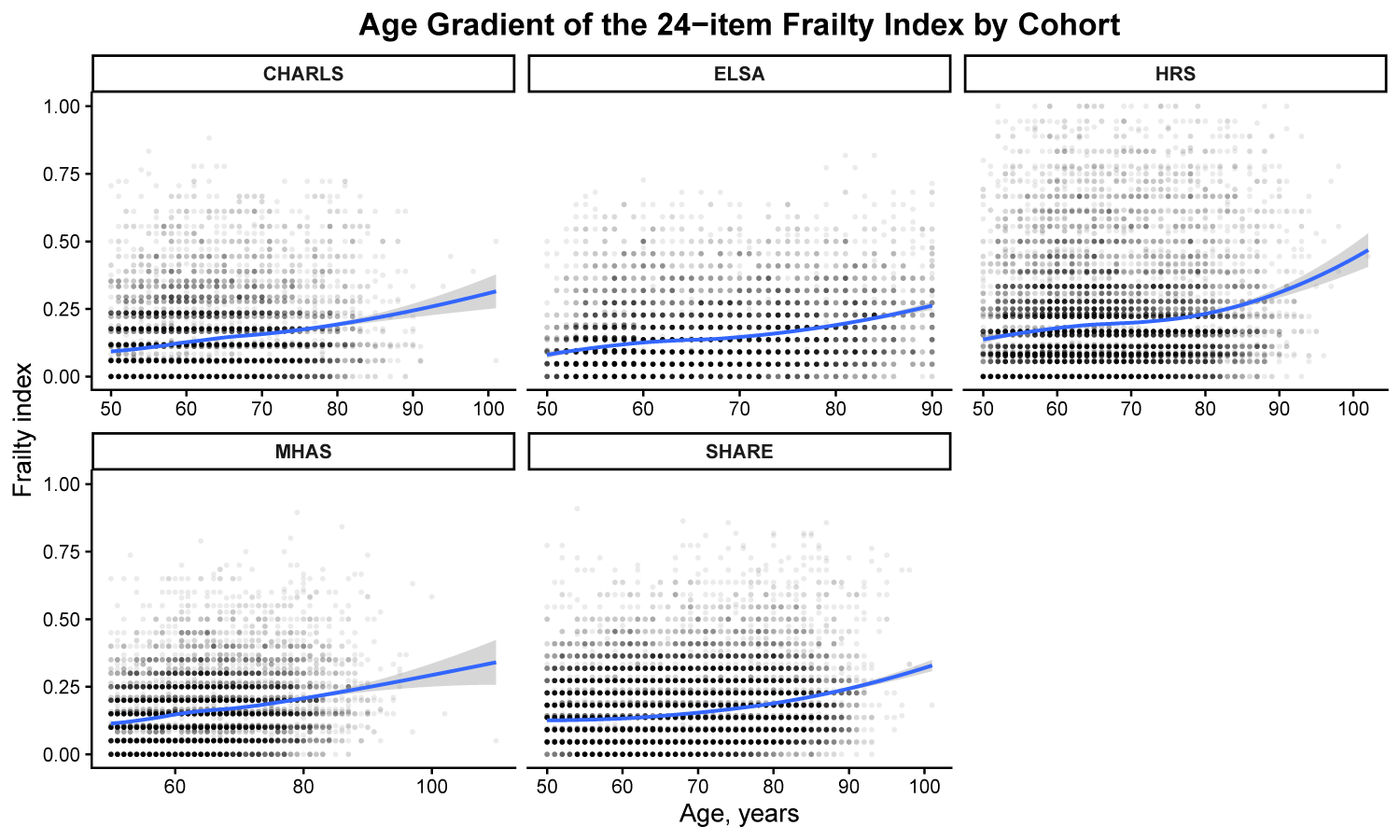

Supplement: Supplementary file 2 [file Image_1.tif]

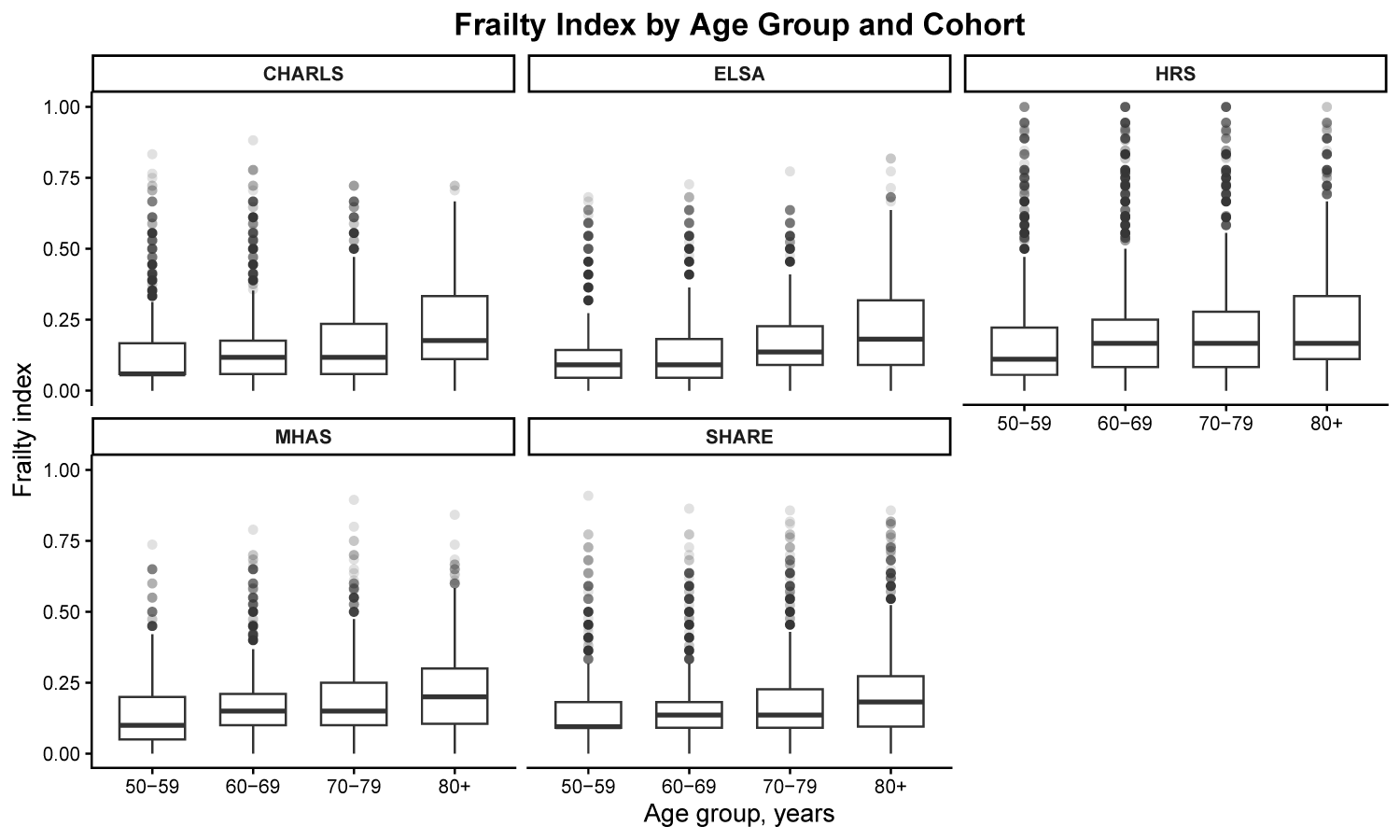

Supplement: Supplementary file 3 [file Image_2.tif]

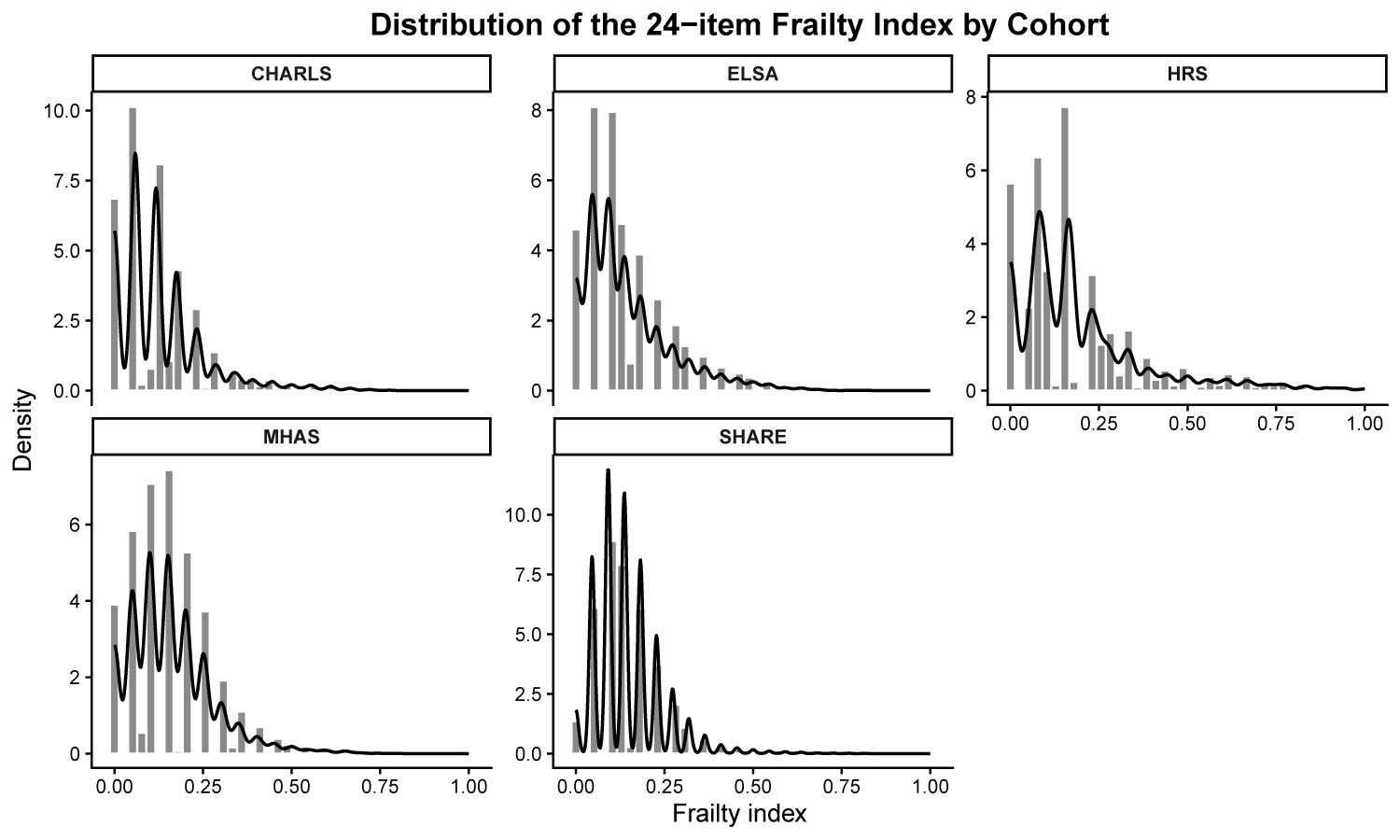

Supplement: Supplementary file 4 [file Image_3.tif]

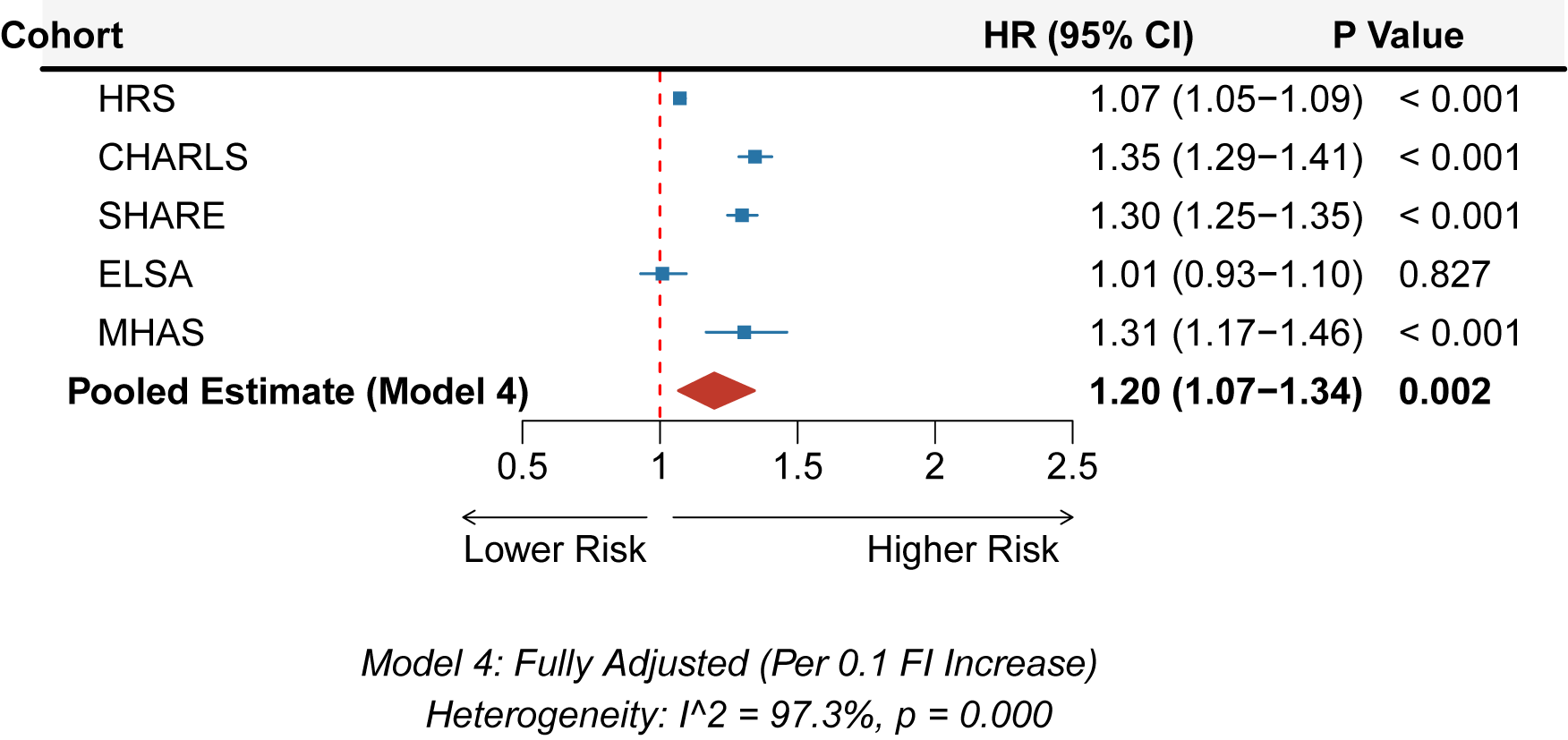

Supplement: Supplementary file 5 [file Image_4.tif]

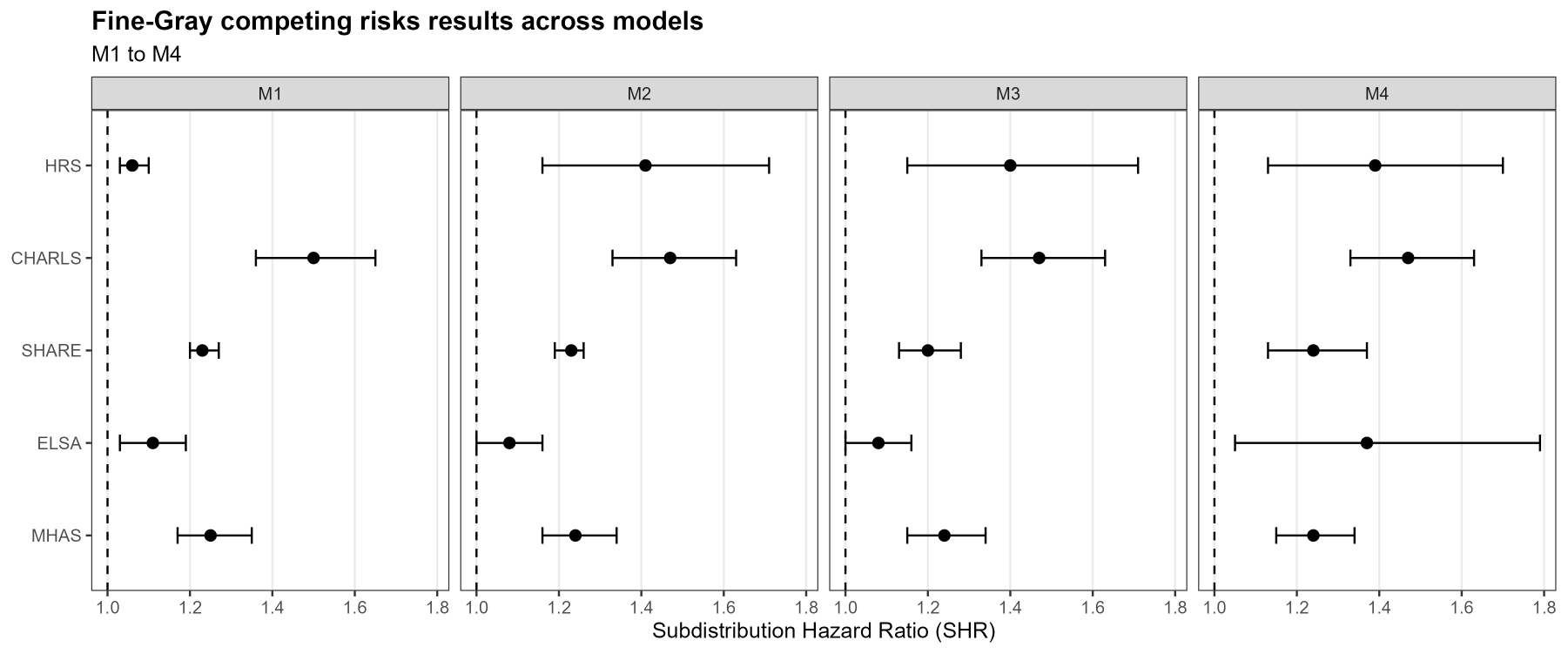

Supplement: Supplementary file 6 [file Image_5.tif]

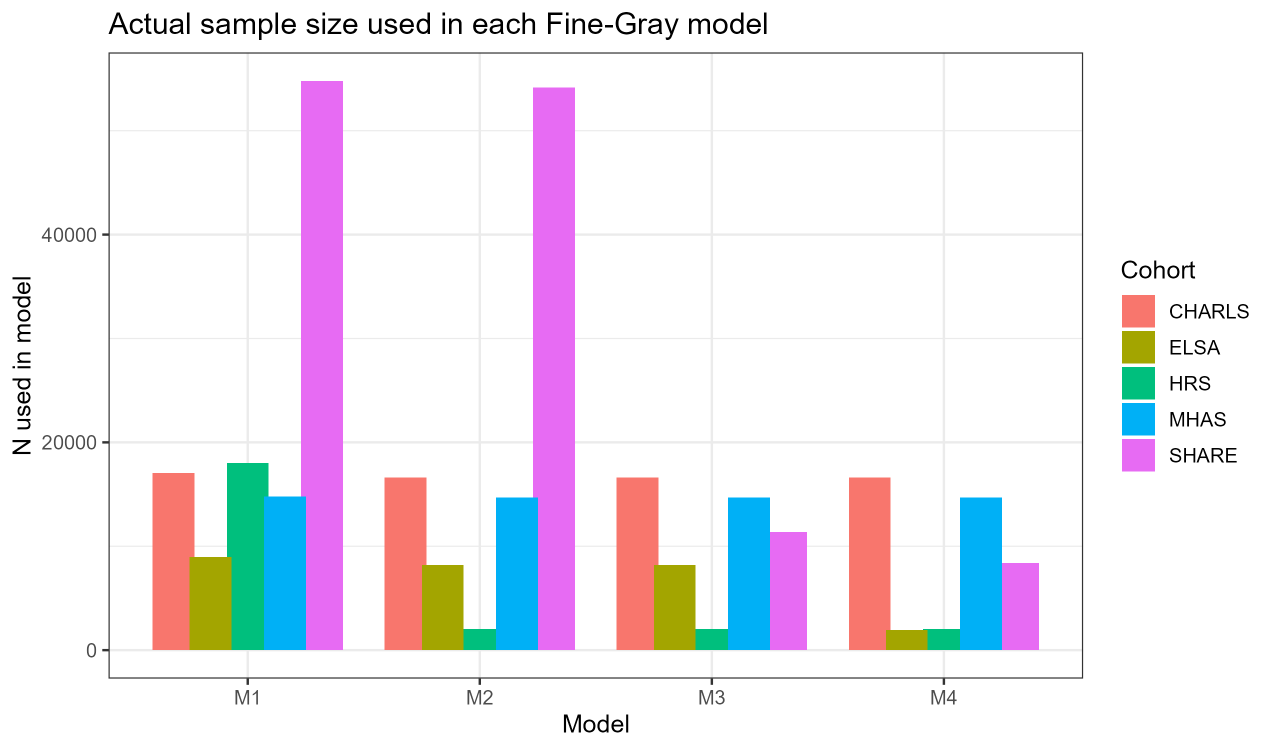

Supplement: Supplementary file 7 [file Image_6.tif]

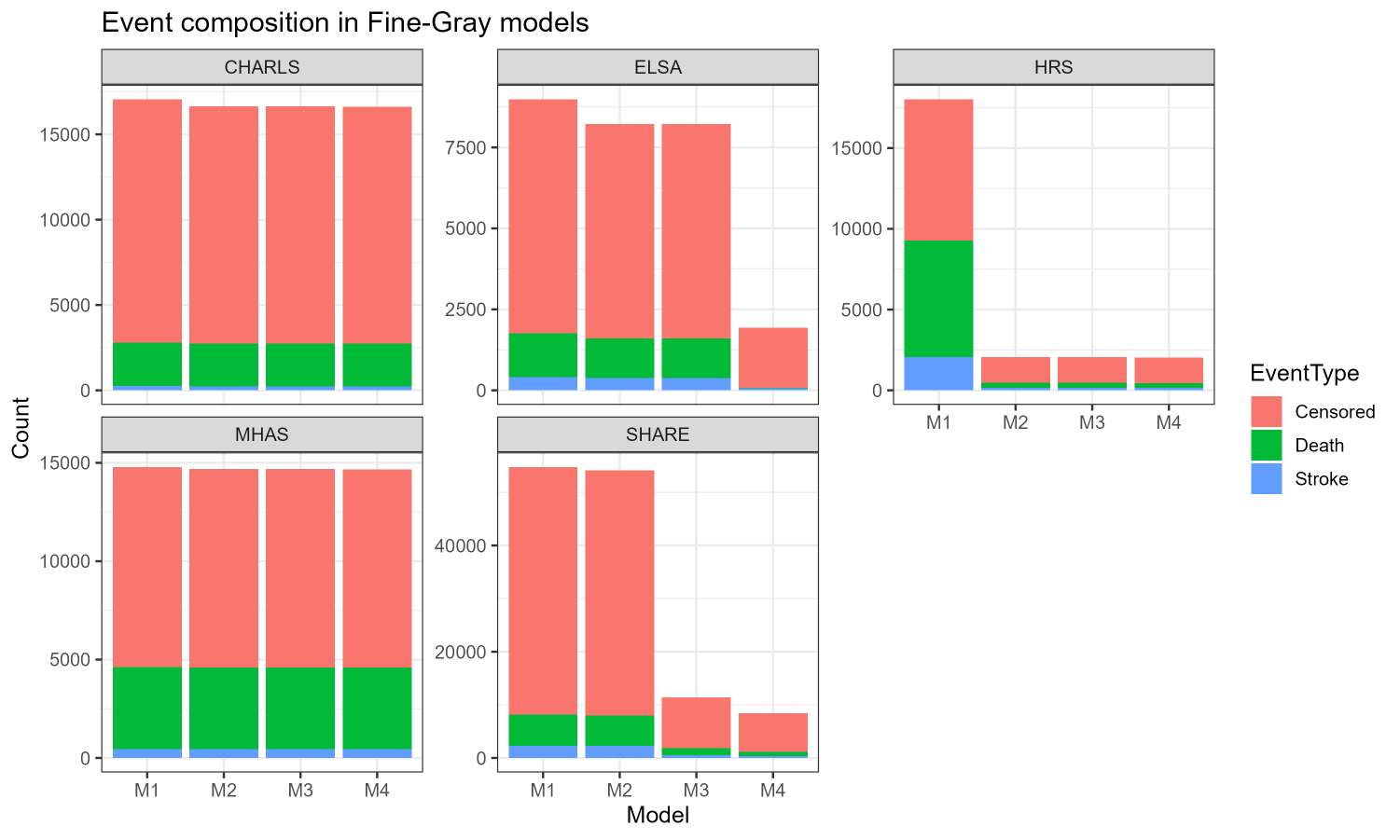

Supplement: Supplementary file 8 [file Image_7.tif]

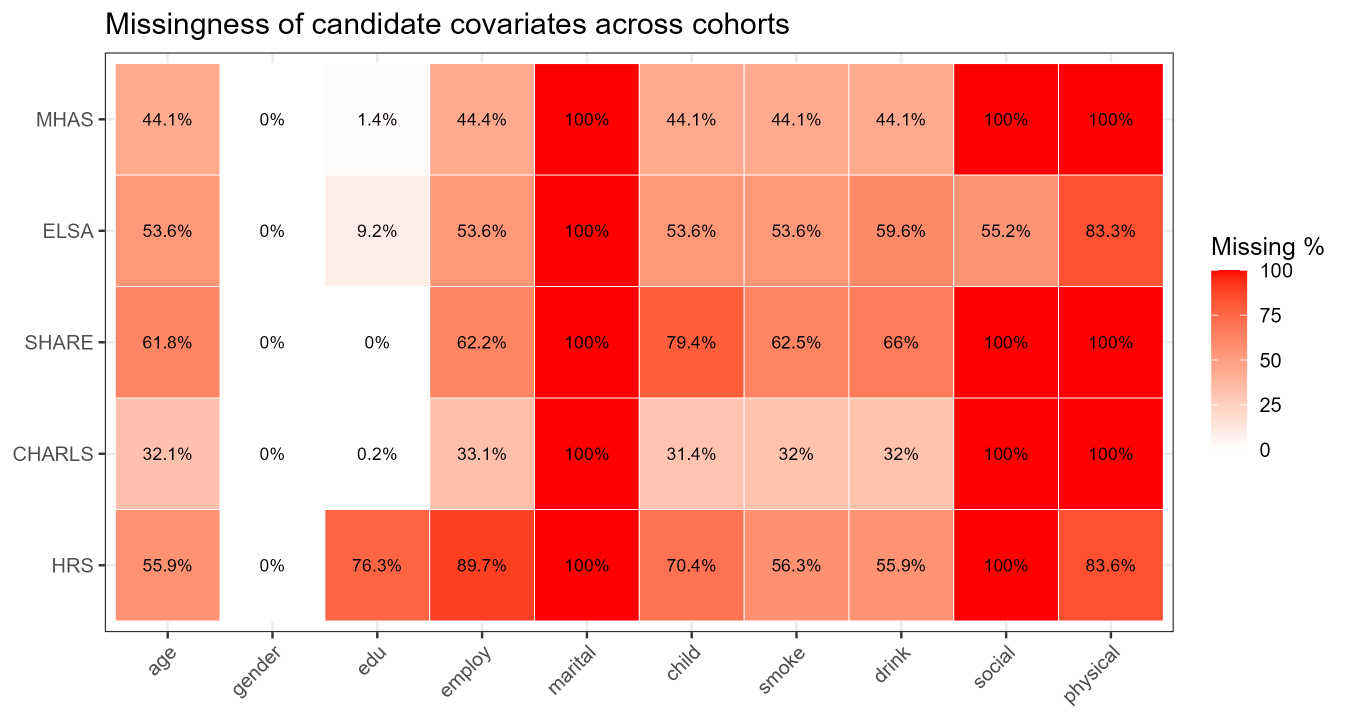

Supplement: Supplementary file 9 [file Image_8.tif]
